# Testing the PPAR hypothesis of tobacco use disorder in humans: A randomized trial of the impact of gemfibrozil (a partial PPARα agonist) in smokers

**DOI:** 10.1371/journal.pone.0201512

**Published:** 2018-09-27

**Authors:** Marie N. S. Gendy, Patricia Di Ciano, William J. Kowalczyk, Sean P. Barrett, Tony P. George, Stephen Heishman, Bernard Le Foll

**Affiliations:** 1 Translational Addiction Research Laboratory, Centre for Addiction and Mental Health, Toronto, Ontario, Canada; 2 Department of Pharmacology, University of Toronto, Toronto, Ontario, Canada; 3 Intramural Research Program, National Institute on Drug Abuse, National Institutes of Health, Baltimore, MD, United States of America; 4 Department of Psychology, Hartwick College, Oneonta, New York, United States of America; 5 Department of Psychology & Neuroscience, Dalhousie University, Halifax, Nova Scotia, Canada; 6 Addictions Division, Campbell Family Mental Health Research Institute, Centre for Addiction and Mental Health, Toronto, Ontario, Canada; 7 Department of Psychiatry, Division of Brain and Therapeutics, University of Toronto, Toronto, Ontario, Canada; 8 Institute of Medical Sciences, University of Toronto, Toronto, Ontario, Canada; 9 Biobehavioural Addictions and Concurrent Disorders Research Laboratory (BACDRL) Centre for Addiction and Mental Health (CAMH), Toronto, Ontario, Canada; 10 Alcohol Research and Treatment Clinic, Addiction Medicine Services, Ambulatory Care and Structured Treatments, Centre for Addiction and Mental Health, Toronto, Ontario, Canada; 11 Department of Family and Community Medicine, University of Toronto, Toronto, Ontario, Canada; Public Library of Science, UNITED KINGDOM

## Abstract

Previous pre-clinical studies demonstrated a promising role of alpha-type peroxisome proliferator-activated receptors (PPARα) agonists in decreasing nicotine self-administration and nicotine-seeking behavior in animals. Our goal was to investigate the potential of gemfibrozil, a PPARα agonist, on reducing tobacco smoking in humans. Methods: This was a double-blind, placebo-controlled, crossover study evaluating the effects of gemfibrozil (1200 mg/day) on smoking in 27 treatment-seeking smokers. The study had two 2-week phases separated by a washout period of at least 1 week. In each phase and after 1 week on medication, participants underwent a lab session where cue reactivity and forced choice paradigms were conducted. Physiological responses and self-reported craving were monitored during the presentation of smoking and neutral cues. In addition, two types of cigarettes were used in the forced choice paradigms: the Nicotinized cigarettes (Nic) and the Denicotinized cigarettes (Denic). The goal of the forced choice was to calculate the percentage of choice of Nic cigarettes while taking gemfibrozil or placebo. The number of quit days was calculated during the two quit attempts weeks (one while taking gemfibrozil and one while taking placebo) of the study. Results: There were no significant differences between gemfibrozil and placebo groups in the percentage of choice of Nic cigarettes, the cue-reactivity (both physiological and subjective measures), or in the number of days of abstinence. Conclusions: Although preclinical studies with PPAR α agonists showed promising results, this preliminary study did not demonstrate positive effect of gemfibrozil on tobacco use and cessation indices.

## Introduction

Every year, six million people die from smoking related disease [1]. Out of 70% of smokers who want to quit, only 4 to 7% are successful [2]. The success rate of the available medications for tobacco use disorder is limited at one year follow-up [3]. Hence, there is a need for new and more effective treatments for nicotine dependence. In this regard, previous studies done with rodents have shown that fatty acid amide hydrolase (FAAH) may be involved in nicotine dependence [4]. For example, administration of a FAAH inhibitor lead to suppression of nicotine-induced conditioned place preference, nicotine self-administration and nicotine-induced increase in dopamine levels in the nucleus accumbens [5, 6]. FAAH inhibition caused an increase in the levels of anandamide (an endogenous cannabinoid), but also elevations of alpha-type peroxisome proliferator-activated receptors (PPARα) endogenous ligands, such as oleoylethanolamide (OEA) and palmitoylethanolamide (PEA) [7]. Therefore, PPARs may be involved in nicotine dependence.

There are three types of PPAR receptors which are: PPAR-α, PPAR-β/δ, and PPAR-γ [8]. PPAR α agonist administration decreased nicotine seeking behavior and nicotine self-administration [9]. In rats given PPARα agonists, nicotine-induced firing of ventral tegmental area neurons and nicotine-induced dopamine release in the nucleus accumbens were decreased [10]. Therefore, PPARα agonists may interfere with the rewarding effects of nicotine, these effects disappeared in the presence of a PPARα antagonist [9]. Together, these studies suggest that PPAR α agonists might serve as a new potential treatment for nicotine addiction.

In humans, Gemfibrozil (Lopid ®), a PPARα agonist was marketed in 1976 and was approved by the FDA and Health Canada to lower the elevated blood triglycerides and cholesterol levels [11–13]. Gemfibrozil is well absorbed orally, has a short half-life, and undergoes extensive hepatic metabolism [14]. 70% of gemfibrozil and its metabolites are excreted in urine [15]. Serious drug-drug interactions can occur with other medications including, HMG-CoA reductase inhibitors, warfarin, and oral hypoglycemic [15]. However, according to previous studies, gemfibrozil is known to have low affinity to the PPAR α receptor [16].

The aim of this study was to investigate the therapeutic potential of gemfibrozil in treating nicotine dependence. It has been proposed that laboratory studies can provide a more cost effective and efficient methods of screening medications for their therapeutic potential [17]. The ability of new medications to attenuate the adverse effects of withdrawal and craving can be evaluated through subjective reports after a period of abstinence [18]. Another way of testing a new smoking cessation drug is to monitor smoking behavior and satisfaction while taking the new medication [17]. In this study, we used a validated screening methodology [19, 20] to conduct a short-term laboratory-based clinical trial with gemfibrozil. We used a within-subject design to increase statistical power. Recruited participants were motivated to quit smoking in the near future. Our primary outcome was the number of abstinence days. In a cue-reactivity test, participants were exposed to either neutral or smoking cues while subjective and physiological measures were taken. During a forced-choice paradigm, participants chose between nicotinized (Nic) or denicotinized (Denic) cigarettes. We hypothesized that gemfibrozil would decrease laboratory measures of nicotine dependence and would increase the number of days of abstinence compared to placebo.

## Materials and methods

### Study design

This was a double-blind, placebo-controlled, crossover design comparing the effects of gemfibrozil to placebo on cigarette smoking in smokers who were motivated to quit smoking. The study had two 2-week phases separated by a washout period of at least 1 week. After an assessment visit, subjects fulfilling our inclusion and exclusion criteria were enrolled in the study. Then, participants received gemfibrozil (1200 mg/day) or placebo for two weeks in a counterbalanced order. At the end of the first week of medication, laboratory measures of cue-elicited craving and forced-choice paradigms occurred. During the second week of medication, participants were asked to stop smoking for the whole week and to report the number of cigarettes smoked daily by phone, i.e. a smoking quit attempt (Table 1). In addition, after completing the two 2-week phases, participants were provided referral information to smoking cessation programs at the Centre for Addiction and Mental Health (CAMH). Participants were contacted by telephone one week after the last medication phase to assess side effects, and then were discharged from the study. The participants were told that they do not have to resume smoking if they have achieved abstinence in the first phase, but they wouldn’t be able to continue in the study (S1 Protocol). The primary outcome of the study was the number of days of abstinence during the smoking quit attempt after one week of treatment. Secondary outcomes were the percentage of choice of puffs from the Nic cigarettes and the subjective and physiological measures during the cue-reactivity session.

**Table 1:** Overview of study design and visits.

Study phase	Activities
**Assessment**	Screening for eligibility
**Medication visit 1**	Attend clinic to receive medication. Medication start day was selected to be at least 7 days before the laboratory test days.
**1**^**st**^ **week of medication**	Smoke normally; take medication (gemfibrozil or placebo). Attend clinic for laboratory tests after taking medication for at least one week. Call in daily.
**2**^**nd**^ **week of medication**	Quit attempt week while taking medication. Attend clinic at the end of the week to assess abstinence. Call in daily.
**Washout week**	No medication for at least a week.
**Medication visit 2**	Attend clinic to receive medication. Medication start day was selected to be at least 7 days before the laboratory test days.
**3**^**rd**^ **week of medication**	Smoke normally; take medication (gemfibrozil or placebo). Attend clinic for laboratory tests after taking medication for at least one week. Call in daily.
**4**^**th**^ **week of medication**	Quit attempt week while taking medication. Attend clinic at the end of the week to assess abstinence. Call in daily.
**1 week follow-up**	Calling the participants to rule out any side effects.

Study procedures and design were approved by CAMH Research Ethics Board (REB) before the conduct of the study (#082–2012). In addition, a written informed consent form approved by CAMH REB was signed by each participant before starting the assessment visit. The study registration ID in the clinical trial.gov is NCT01876810). Our hypothesis was that gemfibrozil would decrease tobacco related cue-reactivity, the number of puffs taken on the Nic cigarette and increase abstinence during the smoking quit attempt. In the absence of human clinical data, we based our hypothesis on preclinical data that suggested an effect size of 0.5. In order to detect a smaller effect size, we aimed to recruit 40 subjects to have sufficient power (>80%) at an alpha level of 0.05, taking into account the variance of effects based on previous human laboratory studies of such effects [21]. An interim analysis was conducted and the study was closed prematurely in December 2015 due to lack of any effects on abstinence. One participant (GEMTO 011) was excluded from the study after randomization due to an initially undisclosed mental illness that was one of the exclusion criteria. The total analyzed number was 27 subjects recruited between April 2014 and September 2015.

### Recruitment (Fig 1)

Participants were recruited through posters, newspaper ads and web-based advertisements such as Craigslist and Kijiji. Inclusion criteria were: 19–65 year old males and females smoking at least 10 cigarettes per day for at least 2 years and intending to quit smoking within the next 3 months. Participants were medically and psychologically healthy; women capable of becoming pregnant agreed to use birth control during the study. Women provided a negative urine pregnancy test at each clinic visit and were not nursing. Participants were excluded if they were attempting to quit smoking or had received treatment for nicotine dependence in the past 3 months. Exclusion criteria also included a history of drug or alcohol dependence within the last 5 years, consumption of more than 15 alcoholic drinks per week on average during the past month, use of any illicit drug more than once per week on average during the past month, any history of or current cardiovascular, hepatic or renal disease, diabetes, and use of psychoactive drugs as revealed by urine toxicology. Recruitment and study visits took place at CAMH, Toronto, Ontario.

**Fig 1:**
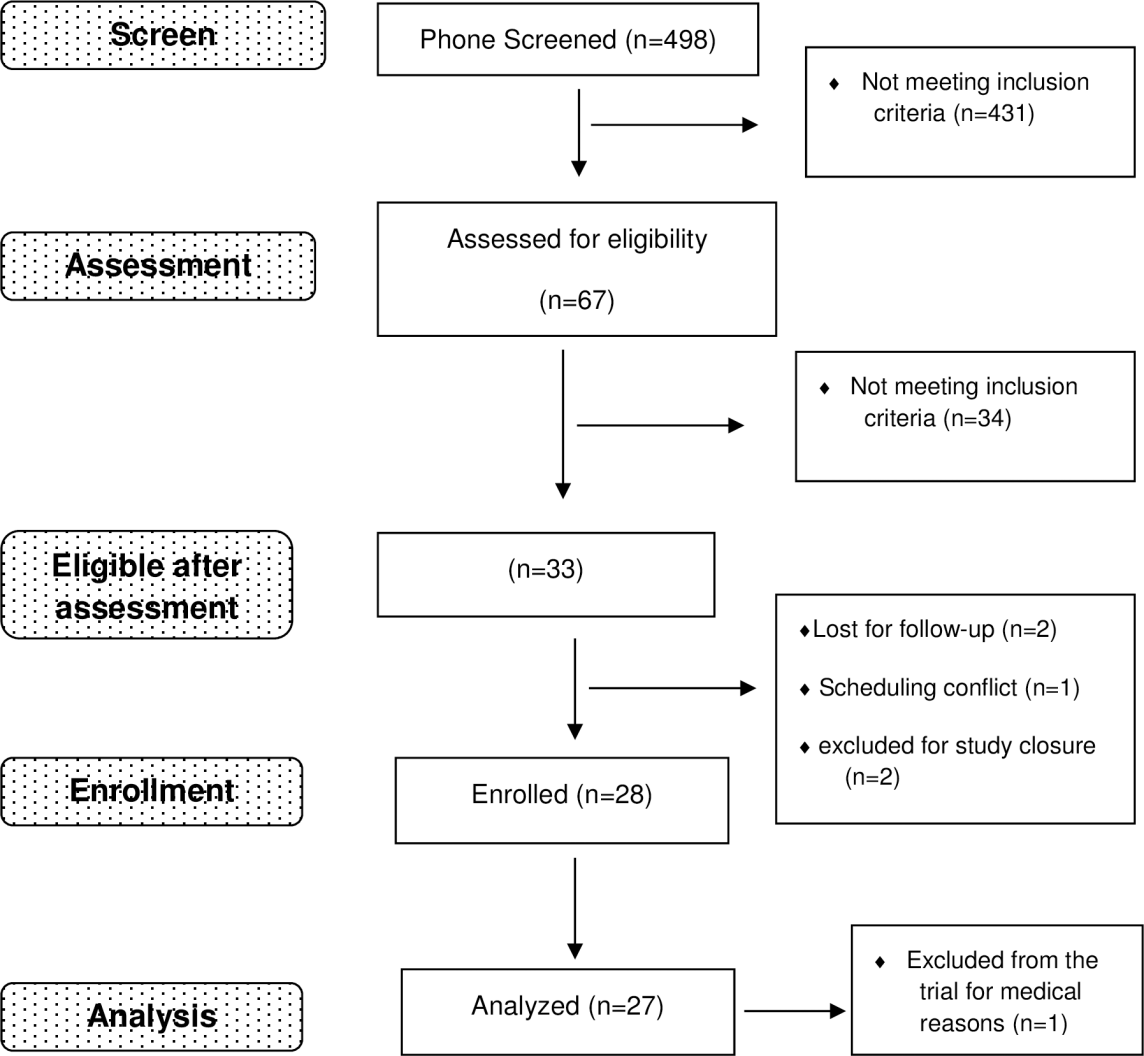
Study flow chart. Participants were recruited through posters, newspaper ads and web-based advertisements such as Craigslist and Kijiji. 498 participants were phone screened, 67 were eligible and came in for in person assessment, 28 participants were enrolled, and the results of 27 participants were analyzed.

### Medication

Gemfibrozil tablets (600 mg) were supplied by Pfizer; Inc. CAMH Research Pharmacy formulated the gemfibrozil and the placebo into capsules and dispensed them to the participants in blister packs. Block randomization was used; block size was 10 participants in counterbalanced order (5 randomly assigned to gemfibrozil for phase #1 and five randomly assigned to placebo for phase #1). The gemfibrozil dose used in this study was that clinically indicated for hypercholesterolemia: 600 mg twice daily taken orally 30 minutes before the morning and evening meals. Although gemfibrozil is considered a safe drug, side effects were monitored on a scale of 0–3 (none, mild, moderate, and severe). Side effects as provided in the Investigator Brochure provided by Pfizer; Inc., included nausea, agitation, nervousness, constipation, dry mouth, fatigue, insomnia, headache, increased appetite and they were monitored in each visit.

#### Medication compliance

Medication compliance was monitored by requiring participants to call a dedicated phone line once a day after taking their second capsule. During the call, they also indicated the number of cigarettes they smoked that day. Participants also returned the blister packs to verify the number of doses taken and breath carbon monoxide (CO) (Pico Model#PP0735123813) was measured at each visit. Smoking abstinence was assessed by self-report and breath CO < 5 ppm at clinic visits [22]

### Laboratory measures and procedures

#### Subjective measures

The following questionnaires were completed at baseline and during the forced-choice and cue-reactivity sessions, as described below: Mood Form [23], Tobacco Craving Questionnaire-Short Form (TCQ-SF) [24], Visual Analog Scale (VAS) for crave a cigarette, urge for a cigarette, positive mood, and negative mood [25], and Minnesota Nicotine Withdrawal Scale (MNWS) [26].

#### Forced choice

The forced-choice paradigm is used to investigate the reinforcing effect of drugs of abuse [27] including nicotine and tobacco [28]. Usually, the study staff presents to the participant two identical drugs, A and B, where one is the active drug and the other is the placebo. After several trials where the drugs are sampled, the participant is asked to choose between both drugs in a series of forced choices. The percentage of choice of the active drug is compared to inactive drug and is viewed as a measure of drug reinforcement [29].

Session began with participants taking four puffs of their preferred-brand cigarette to standardize the time from last nicotine exposure. Participants were asked to complete Mood Form, TCQ-SF, MNWS, and VAS at baseline. Then, they were asked to relax for 30 min listening to music or reading. Four exposure trials followed that were separated by 30 min of relaxation. In each exposure trial, participants took four puffs of a Nic (A) or Denic (B) cigarette in the order of ABAB or BABA. Cigarettes were color-coded, and participants rated each cigarette after the puffs using the modified cigarette evaluation questionnaire [30]. Participants then began four choice trials separated by 30 min of relaxation. In each trial, participants chose any combination of 4 puffs from the two cigarettes. For the Nic cigarettes, commercially-available cigarettes were used (Players Rich). Denic cigarettes were Quest® 3 (Vector Tobacco Inc.) cigarettes that delivered less than 0.05 mg nicotine.

#### Cue reactivity

Cue reactivity is a paradigm used in multiple studies to predict dependence and potential relapse [31–33]. Participants’ physiological responses to different cues (smoking or neutral) such as heart rate, skin temperature, blood pressure and skin conductance are recorded through the session [32, 34]. Session started with participants taking four puffs of their preferred-brand cigarette to standardize the time from last nicotine exposure. Participants were then seated in a comfortable chair and completed the baseline Mood Form, TCQ-SF, VAS, and MNWS. Biopac electronic device (#INI14020000901) was used to record heart rate, blood pressure, skin conductance, and skin temperature during the entire session. The smoking cue was a pack of cigarettes and a lighter. Participants were instructed to light the cigarette without puffing and hold it for 30 sec while the physiological recordings were measured. Then the participant was asked to extinguish the cigarette. The neutral cue was an unsharpened pencil, a notepad, and a sharpener. Participants were instructed to sharpen the pencil and hold it as if writing for 30 sec. Participants completed the TCQ-SF, Mood Form, and VAS forms during the cue, and 15 and 30 min after cue presentation.

### Data analysis

Cue reactivity subjective and physiological measures data were analyzed using the linear Mixed Model for Repeated Measures (MMRM). We defined subjects as clusters and then fitted a random intercept for every subject. Treatment (gemfibrozil; placebo), cue type (smoking; neutral), and period (before, during, after cue) were fixed effects. We also controlled for medication sequence. Forced choice data were analyzed using generalized estimating equation with a binary logistic model that uses logit link. We controlled for the medication sequence, the cigarette type randomization, and for the period of choice (1^st^ and 2^nd^ study phases). The mean number of choice was multiplied by 100 to calculate the percentage of Nic puffs choice. The mean (±SEM) number of days of abstinence was as calculated for each participant during quit attempts for both treatments. The effects of gemfibrozil or placebo on the number of cigarettes smoked each day for 7 days during the quit attempt week was analyzed with MMRM where we controlled for the sequence of medication and the period of abstinence (baseline, 1^st^ study phase, 2^nd^ study phase). For all analyses, results were considered significant at *p* < 0.05 (SPSS version 21.0).

## Results

Participants (n = 27) were 10 females and 17 males; 52% were White, 15% were Black, 7.5% were Asian, and the remainder participants were of mixed race. Mean (±SD) age of the participants was 43 (±12) years, and mean years of education after high school was 2.4 (±2.2) years. Mean (±SD) cigarettes/day was 18.8 (±7) and participants smoked for 21.2 (±11.6) years at baseline. The Fagerstrom Test for Nicotine Dependence (FTND), which is a reliable, self-administered 6-item questionnaire [35], was measured at screening; mean (±SD) FTND was 5.4 (±1.7). Mean (±SD) baseline smoking contemplation ladder score, as an indicator for motivation to quit was 7.2 (±2.9).

During the study, gemfibrozil was well tolerated, with mild to moderate side effects reported as follows: participants taking gemfibrozil in the 1st phase then placebo (n = 14) reported headache (n = 3), change in appetite (n = 2) and stomach upset (n = 3). Participants taking placebo in the first phase then gemfibrozil (n = 13), reported change in appetite (n = 4) and stomach upset (n = 2). All side effects were resolved at following up. No participants reported serious side effects.

### Days of abstinence

**Fig 2:**
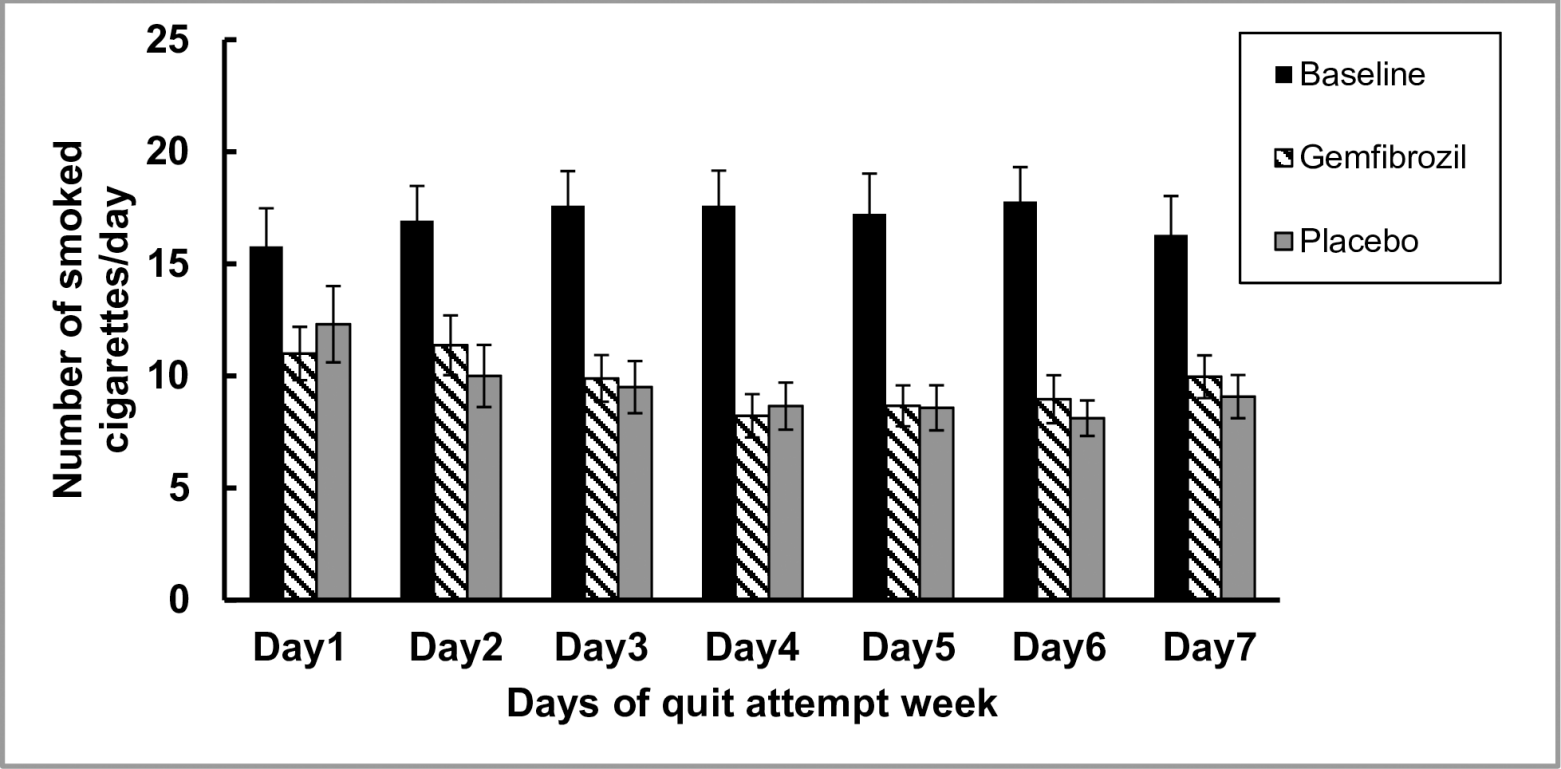
Number of cigarettes/day during quit attempt weeks. Self-reported number of smoked cigarettes per day for 7 days at baseline (black bars), during the quit attempt week taking gemfibrozil (pattern bars) and during the quit attempt week taking placebo (grey bars). After comparing the effects of both treatments using a linear mixed model, the number of smoked cigarettes/day was significantly higher during baseline compared to both treatments (< 0.001). No significant difference was detected between gemfibrozil and placebo groups.

No significant difference was found between both treatments with respect to number of days of self-reported abstinence during both quit attempt weeks: mean (±SEM) = 0.2 (±0.1) day for gemfibrozil versus 0 day for placebo. The average number of smoked cigarettes for 1 week at baseline mean (±SEM) was significantly higher than the average number of smoked cigarettes during either gemfibrozil or placebo quit attempt week: mean (±SEM) at baseline: 16.8 (±1.1); F (2,54) = 31.8; p = (< 0.001); 95% CI [14.5, 19]; (gemfibrozil 9.8 (±1.1); 95% CI [7.5, 12] and placebo 9.6 (±1.1); 95% CI [7.3, 11.9]); respectively. Bonferroni adjustment for multiple comparisons revealed no significance between both treatments (mean diff. = 0.2 (±1.03); 95% CI [-2.3, 2.7]; p = 1) (Fig 2).

### Forced choice

**Fig 3:**
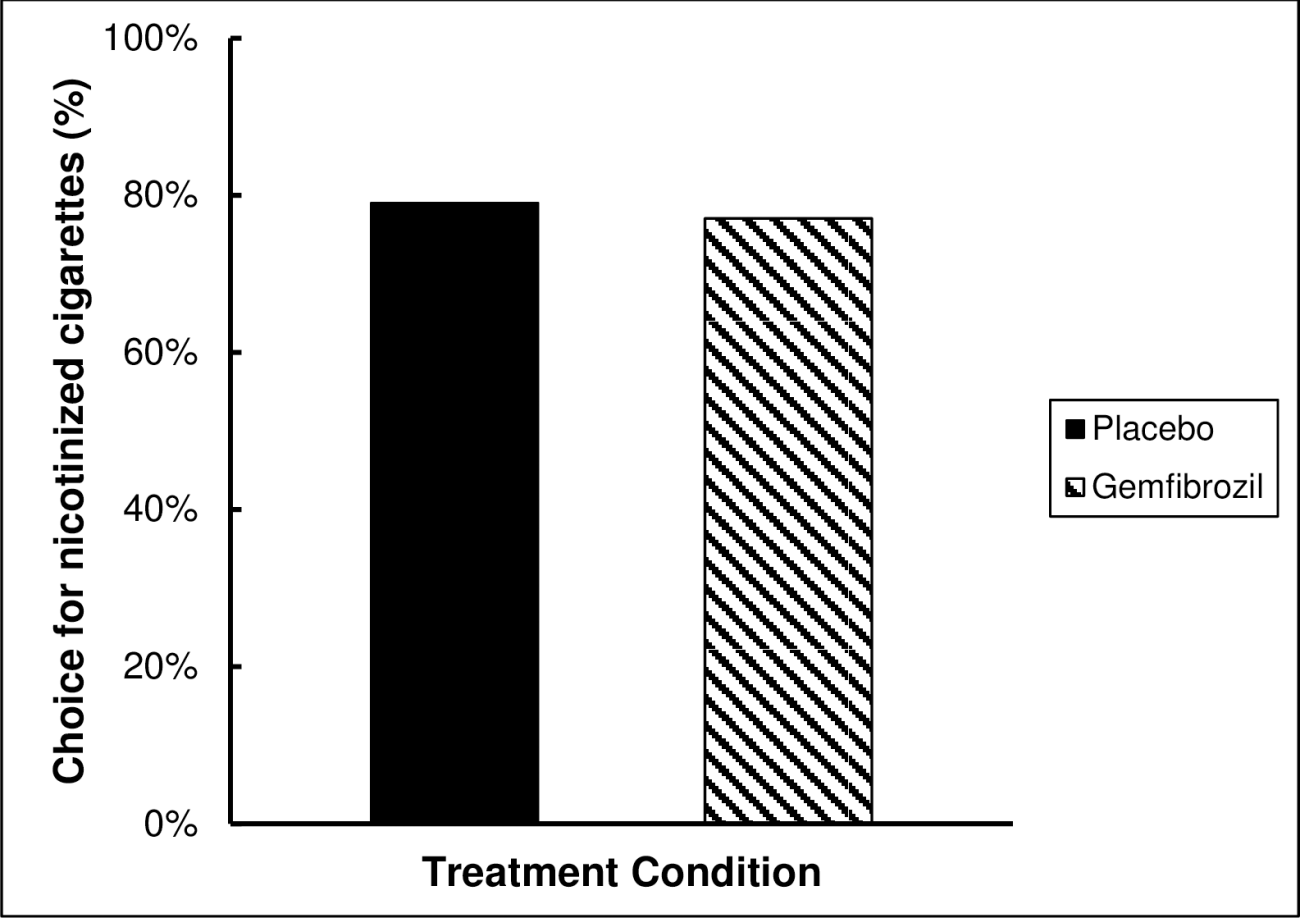
Nicotinized cigarette percent choice: Percent choice of Nicotinized cigarette puffs during the forced choice paradigm showing no significant difference between gemfibrozil (77%) and placebo (79%).

The effect of both treatments on Nic puff choice was calculated using generalized estimating equation. No significant difference was found between treatments [estimated marginal means (±SEM): 0.77 (±0.04); 95% CI [0.68, 0.84]; and 0.79 (±0.04); 95% CI [0.7, 0.86] for gemfibrozil and placebo; respectively p = 0.7 (Fig 3).

### Smoking cue reactivity (physiological measures)

Linear mixed model for repeated measures (Drug x Time x Cue) revealed no drug effect. However, a significant effect of cue was observed for skin conductance, where values were higher in response to smoking cue with mean difference (mean diff.) = 0.26 (±0.12), F (1,390) = 4.7; p = 0.031; 95% CI [0.02, 0.5]. For heart rate, there was a significant decrease over time for both treatments, F (3,390) = 5.9; p = 0.001. Bonferroni adjustment for multiple comparisons revealed a significant decrease after 30 minutes of cue presentation than at cue, with mean diff. = -2.6 (±0.8); F (3,390) = 5.9; p = 0.006; 95% CI [-4.7, -0.5] and from before cue, with mean diff. = -3.1 (±0.8); F (3,390) = 5.9; p = 0.001; 95% CI [-5.1,-0.9]. Skin temperature showed a cue effect where temperature was lower in smoking cue versus neutral cue, mean diff. = -0.2(±0.1); F (1,390) = 5.6; p = 0.02; 95% CI [-0.4, -0.04]. We could not interpret blood pressure data with many missing periods of recording due to technical issues.

### Smoking cue reactivity (subjective measures)

Linear mixed model for repeated measures (Drug x Time x Cue) revealed no drug effect (S1 Dataset). However, a significant decrease of VAS positive mood over time, F (2,286) = 5.8; p = 0.003 was observed. Bonferroni adjustment for multiple comparisons showed that VAS positive at cue and after cue were lower than before cue for both treatments (with mean diff. = -3.3 (±1.2); 95% CI [-6.2,-0.4]; p = 0.02) and (-3.8 (±1.2); 95% CI [-6.7, -0.9]; p = 0.005); respectively. VAS urge showed a significant increase over time, F (2,286) = 19.3; p < 0.001. Bonferroni adjustment for multiple comparisons showed that VAS urge was higher at and after cue than before for both treatments (with mean diff. = 8.9 (±2.4); 95% CI [3.2, 14.8]; p = 0.001) and (14.9 (±2.4); 95% CI [9.1, 20.7]; p < 0.001); respectively. There was a significant increase in VAS craving over time for both treatments, F (2, 286) = 10.3; p < 0.001. Bonferroni adjustment for multiple comparisons showed that VAS craving at and after cue were higher than before (with mean diff. = 7.2 (±2.7); 95% CI [0.7, 13.7]; p = 0.023) and (12.2 (±2.7); 95% CI [5.7, 18.7]; p < 0.001); respectively. Mood positive questionnaire showed a significant decrease over time where after cue was lower than before cue (with mean diff. = -0.9 (±0.3); 95% CI [-1.7, -0.1]; F (2,286) = 4.1; p = 0.025). Also, TCQ factor 1for expectancy and TCQ factor 2 for emotionality showed a significant increase over time for both treatments, where at and after cue scores were higher than before cue in both questionnaires [Bonferroni adjustment for multiple comparisons: (TCQ1at cue mean diff. = 0.8 (± 0.3); 95% CI [0.1, 1.5]; F (2,286) = 6.5; p = 0.028); (TCQ1 after cue mean diff. = 1 (± 0.3); 95% CI [0.3, 1.7]; F (2,286) = 6.5; p = 0.002); (TCQ2 at cue mean diff. = 1.5 (±0.4); 95% CI [0.4, 2.5]; F (2,286) = 10.4; p = 0.002); (TCQ2 after cue mean diff. = 1.9 (±0.4); 95% CI [0.8, 2.9]; F (2,286) = 10.4; p < 0.001).

## Discussion

Gemfibrozil did not increase the number of days of smoking abstinence during the quit attempt week versus placebo. Percent choice of Nic cigarettes during the forced-choice test was the same while taking gemfibrozil or placebo. Similarly, the effects of gemfibrozil were not different from placebo on physiological and subjective measures during smoking cue reactivity.

Our primary outcome was the number of smoking abstinence days. In this study, participants tried to quit smoking during both quit attempt weeks as shown by the decrease in the number of cigarettes smoked per day during baseline and during the quit attempt week. However, it is surprising that the subjects could not abstain longer than one day. In previous studies the mean quit days in placebo groups could reach 1.9 days [36] The explanation of this difference could be that the participants knew that they could try to quit smoking but there will be a second week of trial (another chance) in the second phase of the study according to the cross-over design. Also, the participants knew that they wouldn’t be excluded from the trial if they do smoke. Although most of smokers who want to quit relapse within their first week of trial [37], an enforcing quitting design could have resulted in more robust commitment [17]. In previous studies, the participants visited the lab every day to monitor their CO during their abstinence. In the present study, the abstinence visit occurred 7 days after the lab day and the participants were instructed to call the designate phone number to record the number of cigarettes during the past 24 hours. It is possible that difference in the design of monitoring every single day of abstinence resulted in that difference in the abstinence days.

During the cue reactivity paradigm, changes in physiological parameters such as heart rate, skin temperature skin conductance and self-reported mood and craving were measured in the presence of a neutral and a smoking-related cue. In general, the interpretation of physiological changes is difficult as these parameters are influenced by factors other than smoking cues, such as stress, hunger and fatigue. In this study, there was no effect of gemfibrozil over placebo with respect to physiological changes and the self-reported responses that might demonstrate its potential use as smoking cessation aid. Significant decreases in heart rate occurred after the presentation of neutral and smoking cues for both gemfibrozil and placebo. Previous studies showed increased heart rate and decreased skin temperature in response to smoking cues [34, 38]. Other studies showed contradictions in physiological responses to different cues [39]. In this study, skin conductance response was greater and skin temperature was significantly lower in response to smoking cue versus neutral cue which agrees with other studies [32, 34]. Self-reported craving and urge to smoke increased over time concomitant with a significant decrease in positive mood. These findings might be explained by the wait between each cue and the frustration of not being able to smoke [40].

During the forced choice paradigm, the number of puffs from Nic cigarettes was calculated for both medications. Previous studies showed that medications reducing nicotine reinforcement effects result in choosing less puffs from Nic cigarettes [29]. In the present study, gemfibrozil didn’t show a significant effect on nicotine reinforcement as the percentage of choice of Nic puffs were very similar between gemfibrozil and placebo.

When this study was conducted in 2014–15, no clinical studies were published on the use of PPARα agonists as aids in smoking cessation. Recently, Perkins et al [36] published a study similar in design to the present one investigating the effect of fenofibrate, another fibrate medication. Consistent with our findings, they reported no effect of fenofibrate over placebo with respect to laboratory measures of nicotine dependence and cue reactivity or days of abstinence during a brief quit attempt.

Regarding the limitations of this study, we were limited to the dose of gemfibrozil prescribed clinically for lipid control which might explain the difference between clinical and preclinical studies. Also, in the current study, the mean (±SD) cigarettes/day was 18.8 (±7) for 21.2 (±11.6) years at baseline. Different smoking pattern would have shown a different response to gemfibrozil. Finally, gemfibrozil was given for two weeks of treatment. This relatively short period could have masked potential effects of the medication.

Translational research aims to export preclinical findings towards human applications. Unfortunately, preclinical data do not always predict the human response to medications. Studies comparing the effect of PPARα ligands, including fibrates, in rodents versus humans showed a species difference in the response of the nuclear receptor. These studies were done on liver hepatocytes and suggested some genetic structural differences between species [41–43]. This species variation may explain a potential difference in the PPAR α receptor response in the brain. Although the three types of the receptor are distributed throughout the body, PPAR α expression in the brain is lower than in other organs [44, 45] and only a small fraction (0.4–0.7%) of PPARα agonist can reach the brain [46, 47]. Therefore, animal studies typically use high drug doses to achieve effects. In humans, the dose of PPARα agonists that reach the brain is not yet known, which could explain the negative results of studies using the dose of medication indicated for lipid control. Recently, Jackson et al 2017 showed that the selective experimental PPAR α agonist WY14643 had better results than fenofibrate for nicotine seeking behavior in a conditioned place preference and nicotine self-administration tests in mice [48]. It is known that fibrates as ligands have low affinity to PPAR α receptors [49] and that the clinical available fibrates show moderate selectivity on PPAR receptors [50]. Therefore, a more potent and selective PPAR α full agonist, once clinically available, might be a potential treatment for smoking cessation.

## Conclusion

Although preclinical studies demonstrated that PPARα agonists might be an aid to smoking cessation, the results of current two clinical trials, the present study and the one by Perkins et al [36], showed no effect of gemfibrozil or fenofibrate, respectively, on laboratory measures of nicotine dependence, cue reactivity, and smoking abstinence during a brief quit attempt.

## Supporting information

S1 CONSORT checklist (DOC)

S1 Dataset Gemfibrozil/Placebo data sheet. (XLSX)

S1 Protocol Gemfibrozil original protocol REB#082–2012. (DOCX)
