# Supplementary material for: Testing the PPAR hypothesis of tobacco use disorder in humans: A randomized trial of the impact of gemfibrozil (a partial PPARα agonist) in smokers
Source: PLoS One. 2018 Sep 27;13(9):e0201512. doi: 10.1371/journal.pone.0201512 (PMC6160014; doi:10.1371/journal.pone.0201512)
Supplement: S1 Protocol — (DOCX) [file pone.0201512.s003.docx]

Initial Screening of Gemfibrozil as a Smoking Cessation Medication

CAMH protocol 082/2012

June,30 2014

Testing the PPAR Hypothesis of Nicotine Dependence in Humans: Gemfibrozil as a Novel Treatment for Tobacco Addiction

Qualified Investigator and Investigator Responsible for Conducting the Trial:

Dr. Bernard Le Foll

Head, Translational Addiction Research Laboratory

33 Russell Stret, Toronto, Ontario, M5S 2S1

Tel: 416 535-8501 x 34772

Sponsor:

Dr. Bernard Le Foll and Centre for Addiction and Mental Health

33 Russell Street, Toronto, Ontario, M5S 2S1

Monitor:

Wanna Mar

Centre for Addiction and Mental Health

455 Spadina Avenue, Toronto, Ontario, M5S 2G8

Clinical Laboratory:

Bell Gateway Building

100 Stokes Street, Toronto, Ontario, M6J 1H4

Pharmacy:

33 Russell Street, Toronto, Ontario, M5S 2S1

Person authorized to sign the protocol and protocol amendments: Dr. Bernard Le Foll

**Table of Contents**

Précis 3

Introduction 4

Study Aims 11

Participants 11

Study Design and Methods 12

Risks and Discomforts 16

Participant Safety Monitoring 18

Outcome Measures 19

Statistical Analysis 19

Human Subjects Protections 21

Benefits 22

Classification of Risk 22

Consent Document and Process 22

Data and Safety Monitoring 23

Blinding and Unblinding…………………………………………………………………………23

Quality Assurance 24

Adverse Event Reporting 24

Alternatives to Participation 24

Confidentiality 24

Conflict of Interest 25

Compensation 25

Criteria for termination of the trial……………………………………………………………….25

Data Handling and Record Keeping……………………………………………………………..25

Investigational Product Accountability..…………………………………………………………26

References 27

Appendix 1 32

Appendix 2 33

Appendix 3 35

Appendix 4 36

Appendix 5 37

Appendix 6 39

Appendix 7 40

Appendix 8 41

Appendix 9 42

Appendix 10 43

Appendix 11 44

Appendix 12 45

Appendix 13 46

Appendix 14 47

Appendix 15 48

Appendix 16 49

Appendix 17……………………………………………………………………………………51

Initial Screening of Gemfibrozil as a Smoking Cessation Medication

**Précis**

Summary

*Objectives*

Animal studies have shown that drugs acting as agonists at alpha-type peroxisome proliferator-activated receptors (PPARα) suppress nicotine self-administration, attenuate relapse to nicotine-seeking behavior in the reinstatement model, and block nicotine-induced neuronal firing and dopamine release in reward pathways of the brain. These results have been demonstrated with synthetic PPARα agonists and with fibrate drugs (clofibrate, fenofibrate), which are used clinically to treat elevated cholesterol and triglycerides levels. Thus, PPARα is a potential target for the treatment of tobacco addiction. This is the first human study to investigate whether a fibrate drug (gemfibrozil, Lopid®) can reduce nicotine reward and aid smokers in becoming tobacco abstinent.

The objectives of this study are:

1. to investigate the effect of gemfibrozil on laboratory measures of nicotine reinforcement and cue-elicited craving
2. to screen for the ability of gemfibrozil to aid smoking abstinence during a brief quit attempt
3. to examine the validity of using laboratory measures of tobacco dependence to predict smoking abstinence and possible gemfibrozil-related increases in smoking abstinence

*Study population*

This outpatient study will be conducted at the Center for Addiction and Mental Health (CAMH) in Toronto, Canada. This site will enroll 40 adult smokers who intend to quit smoking in the next 3 months.

*Design*

The study is a double-blind, placebo-controlled, crossover design comparing the effects of gemfibrozil and placebo. The study will comprise two 2-week medication phases with a washout period of at least one week. At the end of the first medication week laboratory measures will be taken and during the second medication week participants will make a quit attempt and abstinence will be assessed.

*Outcome Measures*

Primary outcome measures include laboratory assessments of nicotine reinforcement and smoking cue reactivity. The measure of nicotine reinforcement is the percentage of nicotine cigarette puffs chosen during a forced-choice task. Measures of cue reactivity include tobacco craving, mood, and autonomic responsivity.

The secondary outcome measure is days of smoking abstinence during the quit-attempt weeks. Abstinence is assessed by self-reports of no smoking and by breath carbon monoxide < 5 ppm on clinic visits. Other assessments of abstinence include self-reported tobacco craving and withdrawal.

**Introduction**

Tobacco smoking is the leading preventable cause of morbidity and mortality in the world, yet about one in five adults in the U.S. (46 million people) continue to smoke daily (Centers for Disease Control 2009). Although 70% of smokers say they want to quit smoking, only 4-7% are successful each year (Fiore et al. 2008). Therefore, there is a pressing need to develop effective medications to treat tobacco dependence.

Preclinical Research

Endogenous cannabinoids, such as anandamide, are ligands for the CB1 receptor, and nicotine has been shown to increase anandamide levels in the limbic forebrain (Gonzalez et al. 2002). Anandamide and other endocannabinoids are degraded by the enzyme fatty acid amide hydrolase (FAAH). FAAH inhibitors, such as URB597, which prolongs the effects of anandamide in brain areas where it is released, have been tested in animal models of addiction. Studies have shown that URB597 suppressed (a) nicotine-induced conditioned place preference, (b) acquisition and escalation of nicotine self-administration, (c) reinstatement of extinguished nicotine seeking, and (d) nicotine-induced increases in dopamine in the nucleus accumbens (Forget et al. 2009; Scherma et al. 2008). Interestingly, FAAH inhibition also increases levels of the non-cannabinoid amides, oleoylethanolamide (OEA) and palmitoylethanolamide (PEA), which are endogenous ligands at alpha-type peroxisome proliferator-activated receptors (PPARα) (Fegley et al. 2005).

PPARα are nuclear receptors that regulate several aspects of lipid metabolism. Recent research suggests that agonists at PPARα modulate α4β2 nicotinic receptors by promoting their phosphorylation by tyrosine kinases (Melis et al. 2008, 2010). As mentioned above, the FAAH inhibitor URB597 blocks rewarding/reinforcing and relapse-inducing effects of nicotine (Forget et al. 2009; Scherma et al. 2008). Because URB597 increases levels of endogenous PPARα ligands OEA and PEA, these effects might be modulated by PPARα.

In a series of recent experiments in rats and squirrel monkeys, Mascia et al. (2011) found that the highly-selective PPARα agonists WY14643 and methOEA significantly decreased ongoing nicotine self-administration and suppressed nicotine-induced reinstatement of extinguished nicotine-seeking behavior. They also found in rats that WY14643 and methOEA decreased nicotine-induced firing of dopaminergic neurons in the ventral tegmental area (VTA) and decreased nicotine-induced release of intracellular dopamine in the nucleus accumbens shell. In both the behavioral and electrophysiological studies, pretreatment with the PPARα antagonist MK886 reversed the effects of WY14643 and methOEA, demonstrating the receptor specificity of these effects (Mascia et al. 2011).

These results demonstrate that PPARα agonists prevent effects of nicotine on reward circuitry in the brain and reduce nicotine-taking and nicotine-seeking in animal behavioral models. These experimental PPARα agonist drugs are not available for human use. However, fibrate medications, such as clofibrate, fenofibrate, and gemfibrozil, are PPARα ligands that have been used clinically for decades in the treatment of high cholesterol and triglyceride levels (Fruchart et al. 1999; Keating 2011). Because of the promising effects of PPARα agonists discussed above, studies have been conducted with clofibrate, a first generation PPARα agonist.

Panlilio et al. (2012) examined the effect of clofibrate on reward-related behavioral, electrophysiological, and neurochemical effects of nicotine in rats and squirrel monkeys. Clofibrate prevented the acquisition of nicotine-taking behavior in naive animals, decreased nicotine taking in experienced animals, and attenuated the relapse-inducing effects of re-exposure to nicotine or nicotine-associated cues after a period of abstinence. In the brain, clofibrate blocked nicotine's effects on neuronal firing in the ventral tegmental area and on dopamine release in the nucleus accumbens shell. These behavioral and electrophysiological effects of clofibrate were reversed by MK886, a PPARα antagonist, indicating that its actions were mediated by PPARα.

These preclinical research findings suggest that fibrate medications might decrease nicotine reward and reinforcement in humans and thus promote smoking cessation. This study will address this hypothesis for the first time in humans using the fibrate drug, gemfibrozil.

Gemfibrozil

Gemfibrozil (Lopid®) is a lipid regulating agent that decreases serum triglycerides and very low density lipoprotein (VLDL) cholesterol and increases high density lipoprotein (HDL) cholesterol. Gemfibrozil has been shown to inhibit peripheral lipolysis and to decrease the hepatic extraction of free fatty acids, thus reducing hepatic triglyceride production. Gemfibrozil inhibits synthesis and increases clearance of VLDL carrier apolipoprotein B, leading to a decrease in VLDL production. Clinically, gemfibrozil is used as an adjunct to dietary restrictions in adults with primary hyperlipidemia. After oral administration, gemfibrozil is well absorbed from the gastrointestinal tract. Peak plasma levels occur in 1-2 hours with a plasma half-life of 1.5 hours following multiple doses. Absorption is significantly increased when gemfibrozil is taken 30 minutes before meals.

Laboratory Model of Tobacco Dependence

Preclinical research has shown that PPARα agonists decrease the reinforcing effects of nicotine and block cue-elicited nicotine-seeking responses in the reinstatement model of relapse (Mascia et al. 2011; Panlilio et al. 2012; Scherma et al. 2008). Thus, we will determine the effects of gemfibrozil on two laboratory measures of tobacco dependence: nicotine reinforcement and cue-elicited craving.

*Drug Reinforcement*

In behavioral terms, a positive reinforcer is defined as any stimulus or event that increases the probability of the behavior that immediately preceded it. The field of behavioral pharmacology has applied this notion of reinforcement to the study of drugs. In a typical study, an animal is trained to perform an arbitrary response (e.g., press a lever) to receive a food pellet, which is a natural reinforcer. The food reinforces the behavioral lever press response, and soon, the animal is pressing the lever at a fast rate in order to receive the food pellets. When the lever pressing response is well learned, the animal undergoes surgery to implant a catheter into its jugular vein. After recovery, the animal is placed back in the chamber where it learned to lever press for food, and the catheter is connected to a pump that can deliver small doses of drug to the animal. Now, when the animal presses the lever, it receives a drug injection, rather than a food pellet. On some test days, the animal receives saline instead of drug. Drugs that function as reinforcers increase the rate of lever responding for drug compared to saline. Drug self-administration studies are considered a primary test of whether a drug functions as a reinforcer. Such studies have shown that virtually all drugs abused by humans are self-administered by animals (Griffiths et al. 1980).

Not surprisingly, nicotine has been shown to function as a reinforcer in self-administration paradigms in both animals and humans (Corrigall 1999; Harvey et al. 2004). We now know that nicotine is the component in tobacco that causes addiction (U.S. Department of Health and Human Services 1988), and thus nicotine reinforces the behavior of smoking that leads to its delivery.

The forced-choice paradigm was developed in the late 1970’s to study the reinforcing effects of drugs in humans. The paradigm was validated in the 1980’s in a series of studies testing stimulants and sedatives (de Wit &Johanson 1987) and has since been used to study the reinforcing effects of nearly all drugs of abuse. In the traditional forced-choice task, participants are given standardized exposure to an active drug dose and placebo, identified as Drug A and Drug B. Drug A and Drug B are designed to look identical. This is followed by a series of forced-choice trials in which the participant chooses between Drug A and B and then ingests the chosen drug. If the active drug is chosen on more trials than placebo or more than chance (50%), then that drug is considered to have reinforcing effects (de Wit & Johanson 1987).

Nicotine and tobacco have been tested in such forced-choice procedures. Perkins et al. (1996) reported that a subgroup of smokers who preferred nicotine nasal spray over placebo spray chose nicotine nasal spray on 74% of choice opportunities after overnight deprivation. Cigarettes have also been tested in this paradigm by having smokers sample a nicotine-containing (Nic) cigarette and a denicotinized (Denic) cigarette, then choosing to take puffs from each cigarette at 30-min intervals for 2 hours. Rukstalis et al. (2005) found that naltrexone, but not bupropion decreased the relative reinforcing effects of smoking Nic compared to Denic cigarettes. At NIDA, colleagues are currently investigating the reinforcing effects of nicotine using a similar forced-choice procedure (personal communication). Participants sample a Nic and Denic cigarette twice for a total of 4 trials. This is followed by 6 choice trials at 20-minute intervals; participants choose any combination of 4 puffs from the two cigarettes at each trial. Preliminary data (n = 28) indicate that participants choose puffs of the Nic cigarette on 80% of trials (*p*< .001 compared to Denic), indicating that the forced-choice procedure yields a robust measure of nicotine’s reinforcing effect in humans (Fig. 1).


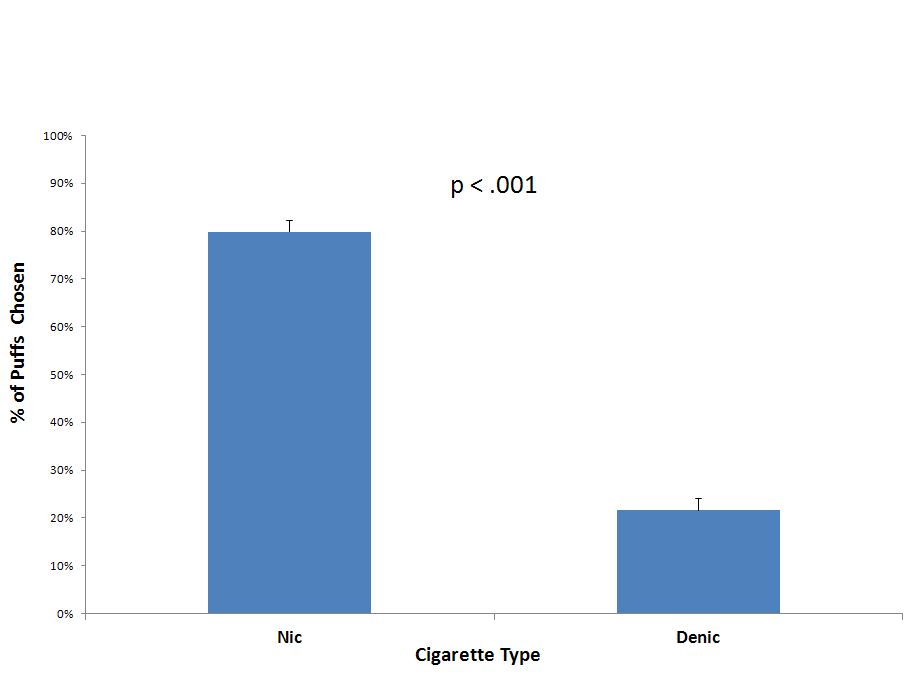


**Fig. 1.**Percentage of puff choice by cigarette type (n = 31). Nic = nicotine-containing cigarette, Denic = denicotinized cigarette. Data from Study 456.

*Cue-elicited craving*

Environmental smoking cues have been studied for decades and are known to play an important role in the maintenance of nicotine addiction and in relapse to smoking. For example, Waters et al. (2003) reported that smokers demonstrated an attentional bias to smoking cues compared with neutral cues. A meta-analysis of cue reactivity studies found increases in self-reported craving and changes in autonomic functioning, such as increases in heart rate and skin conductance, when smokers were exposed to smoking cues compared with neutral cues (Carter & Tiffany 1999). For example, viewing pictures of smokers and smoking paraphernalia or watching another person smoke a cigarette can reliably increase self-reported tobacco craving and cardiovascular measures (Drobes& Tiffany 1997; Niaura et al. 1992; Sayette&Hufford 1994).

A number of studies using imagery scripts to elicit drug craving have been conducted (Heishman et al. 2004, 2006, 2010; Lee et al. 2007; Singleton et al. 2002, 2003; Taylor et al. 1999). In these studies, participants are instructed to actively imagine themselves in an audiotaped scripted scene that involves smoking, such as a party, or a neutral scene and to rate their level of tobacco craving. Such imaginal manipulations reliably increase self-reported craving and autonomic measures, such as heart rate and skin conductance. A recent study compared the ability of *in vivo* smoking cues (having the participant hold a lit cigarette) and imagery scripts describing smoking situations to elicit self-reported tobacco craving and autonomic responses (Heishman et al. 2010). Both cues and imagery scripts increased craving compared to the neutral conditions; however, cues produced a more robust craving response than imagery scripts, especially in the nondeprived condition (Fig. 2). Additionally, in an ongoing study at NIDA (personal communication), significant effects of cues on self-reported cigarette craving and urge to smoke have been observed (Fig. 3). Thus, in this study, we plan to use *in vivo* smoking cues to elicit tobacco craving.

**Fig. 2** Effect of smoking cues and smoking imagery by sex on selected craving, mood, and physiological measures. Graphs on left show data after 12 hr of tobacco deprivation, and graphs on right show data during ad libitum smoking. Each column represents the mean ± SEM of 30 male or 30 female tobacco-dependent smokers. Post hoc comparisons: **p*< .05, ***p*< .01, ****p*< .001.


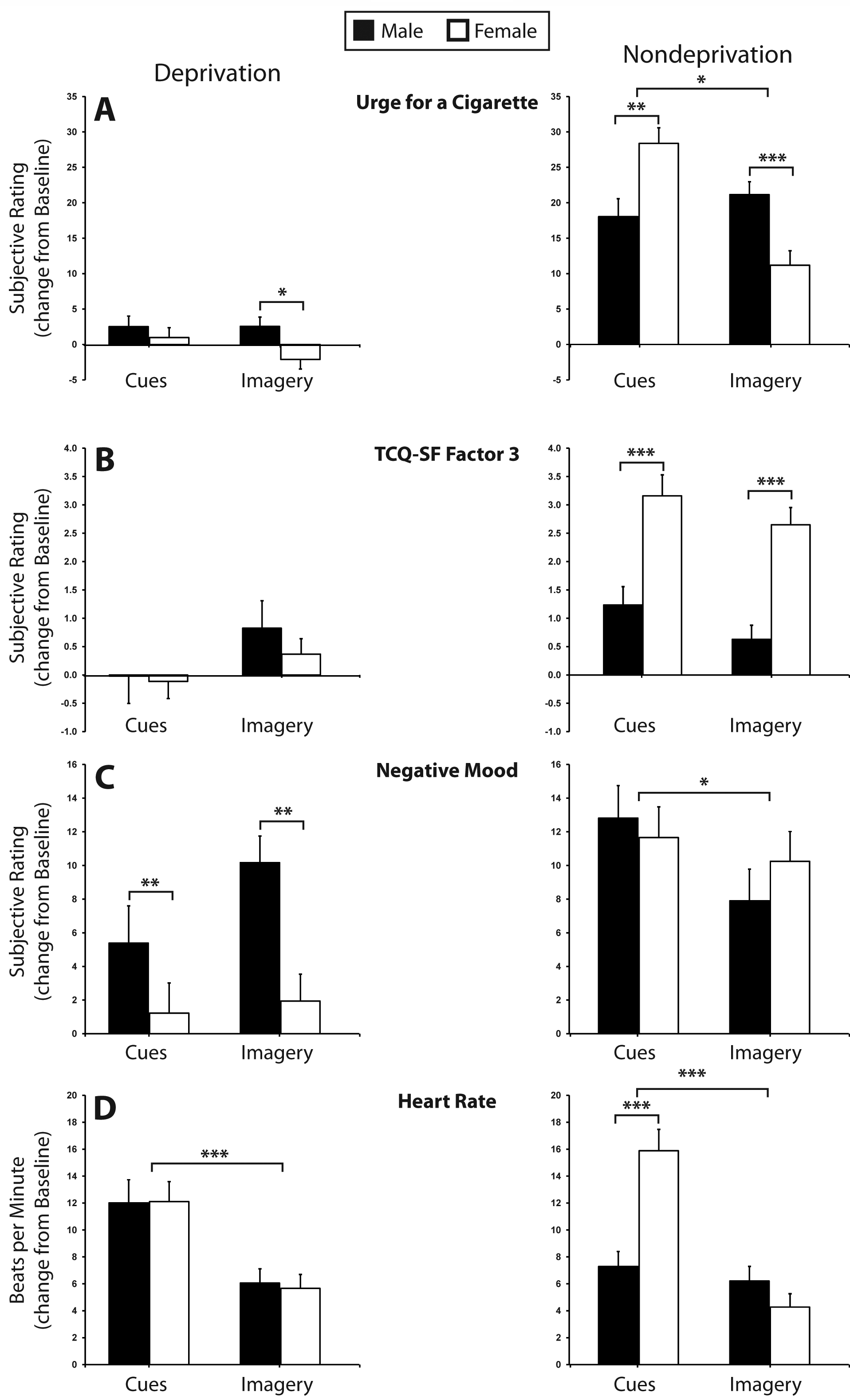

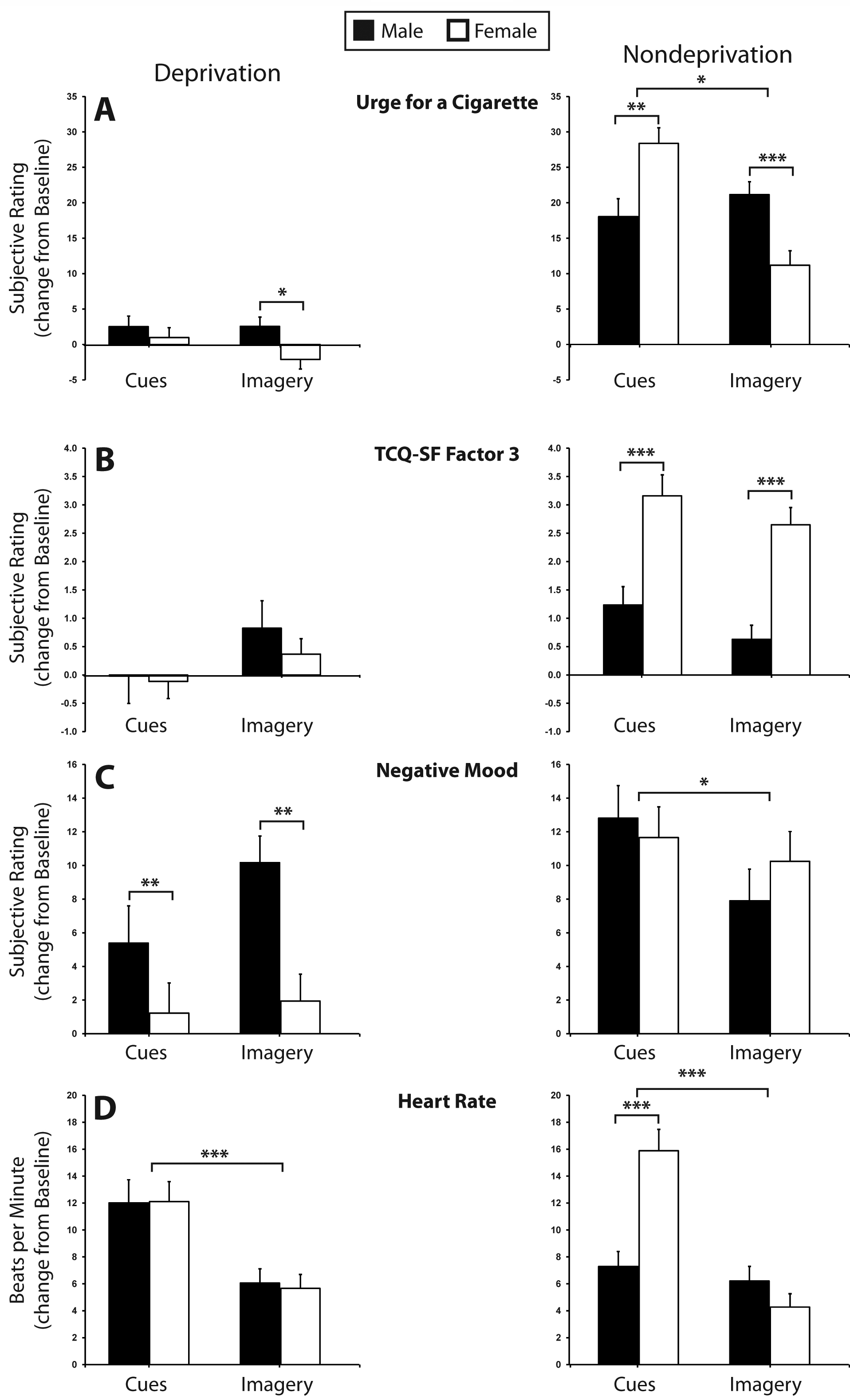

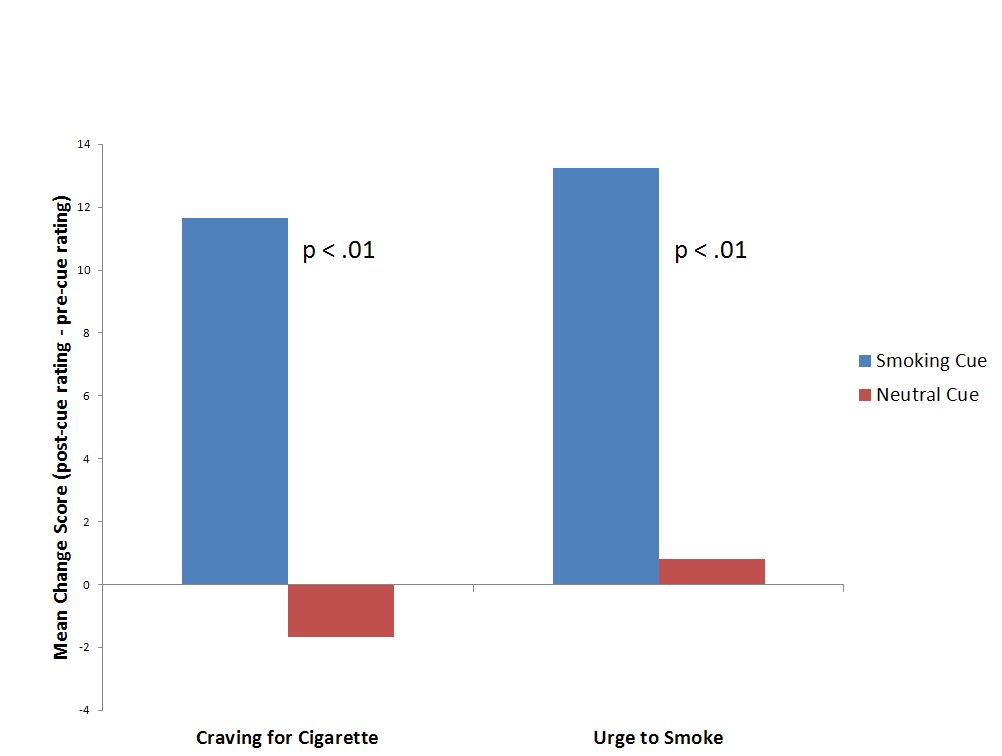


**Fig. 3.**Change from pre- to post- exposure of smoking and neutral cues in visual analog ratings of cigarette craving and urge to smoke (n = 30). From NIDA Study 456.

Brief Assessment of Smoking Abstinence

Up to now, the only valid approach to test the efficacy of a new medication to treat tobacco dependence has been a clinical trial. Such trials typically require large numbers of participants, take months or years to complete, and are expensive. As a result, they are not practical as an initial test of a medication’s efficacy. Recently, Perkins and colleagues (Perkins et al. 2006, 2008, 2010a) developed a new methodology to determine the efficacy of potential medications to treat tobacco dependence. In addition to the impracticality of clinical trials mentioned above, the need for a new screening methodology arose from the realization that current short-term studies that assess the ability of medications to relieve abstinence-induced withdrawal or to decrease the reinforcing effects of smoking do not reliably predict treatment efficacy (Perkins et al. 2006). For example, although nicotine replacement therapy (NRT, patch or gum), bupropion (Zyban®), and varenicline (Champix®) are FDA and Health Canada-approved medications with known efficacy for smoking cessation (Fiore et al. 2008), they have not reliably reduced nicotine withdrawal symptoms or ad libitum smoking in numerous short-term laboratory studies (Perkins et al. 2006, 2010b).

One possible reason that short-term medication studies have not predicted clinical efficacy is that most studies were conducted with smokers not interested in quitting. This lack of motivation to quit smoking would be exclusionary for a clinical trial and thus likely confounded the results of many short-term screening studies. These studies also typically exposed participants to drug for brief durations and assessed withdrawal or craving rather than tobacco abstinence. Thus, Perkins et al. (2006) proposed the following screening methodology: (a) recruitment of smokers planning to quit in the near future, (b) administration of drug for an extended duration, and (c) assessment of tobacco abstinence in participants’ natural environments. Such studies would also retain the advantages of the typical screening study, including brief duration and use of a within-subjects design, which allows increased statistical power with a relatively small sample size.

Perkins et al. (2008, 2010a) conducted two studies to validate this new screening methodology, one using nicotine patch and the other varenicline. In both studies, smokers with high and low intrinsic motivation to quit were recruited. Additionally, half of each group was randomly assigned to receive either $12 per day for maintaining tobacco abstinence or no reinforcement. Using a crossover (within-subjects) design, participants were randomized to receive either active medication first followed by placebo or placebo first followed by active medication. Participants attempted to quit smoking for one week while on medication and placebo. Perkins et al. (2008) reported that nicotine patch doubled days of tobacco abstinence in smokers with high motivation to quit compared to placebo (2.0 ± 0.3 days for nicotine vs. 1.1 ± 0.2 days for placebo). Similarly, Perkins et al. (2010a) showed that varenicline increased days of abstinence in smokers interested in quitting compared to placebo (2.6 ± 0.3 days for varenicline vs. 1.5 ± 0.2 days for placebo). These data indicate that even on medication, participants were not tobacco abstinent on the majority of days, mirroring high relapse rates during the first week of a quit attempt (Fiore et al. 2008). In the varenicline study, Perkins et al. (2010a) found that among those who initiated abstinence during the quit week, 70% on varenicline and 88% on placebo relapsed. Thus, this 1-week quit attempt appears to be a valid model of real-world abstinence success.

Mediators of Treatment Efficacy

Many laboratory studies have examined the ability of medications to either suppress symptoms of tobacco withdrawal, including craving, or reduce the reinforcing effects of smoking. Such studies might identify mediators of a medication’s efficacy, which could advance the development of other medications that target similar or alternate mechanisms. The importance of laboratory models of addiction has been reviewed (Koob 2008; Lerman et al. 2007), but, as outlined above, such models have limited validity in predicting relevant clinical outcomes, such as tobacco abstinence. One laboratory model, cue reactivity has received much attention from tobacco addiction researchers. The act of smoking becomes conditioned to a variety of environmental cues, such that certain cues can reliably evoke craving and relapse. However, there seems to be little predictive association between cue-elicited craving in the laboratory and relapse to smoking in the real world (Abrams et al. 1988; Niaura et al. 1989; Perkins 2009, 2012).

Despite the lack of association found in previous research, the screening methodology developed by Perkins et al. (2006, 2008, 2010a) provides a unique opportunity to examine potential mechanisms of treatment efficacy. Participants can complete questionnaires on multiple occasions reporting on a variety of state measures, including withdrawal, craving, mood, smoking satisfaction, and medication effects. Behavioral measures can be assessed, such as cigarettes smoked per day, time of first cigarette, and changes in sleeping and eating patterns. Laboratory sessions can also be scheduled to measure responses to smoking cues, nicotine reinforcement, and cognitive performance. Over time with a large sample, genotype-phenotype associations can move us toward individualized treatment approaches. This more fine-grained measurement of laboratory responses may be correlated with assessments of real-world tobacco abstinence within each participant. Whereas previous research has failed, this screening technique provides a novel opportunity to examine these associations, so that valid laboratory predictors of smoking abstinence can be elucidated.

Considerations of a Crossover Design

The primary advantage of using a crossover or within-subjects design is efficiency. Greater statistical power can be achieved with a smaller sample size compared with between-subjects or parallel-groups designs, which require more subjects to overcome large inter-subject variability. Additionally, because each participant serves as his or her own control in a crossover study, matching on sociodemographic and smoking history variables that might confound outcome measures is not necessary.

Crossover designs are best suited to short-term outcome studies of chronic diseases that persist throughout active drug and placebo phases (Sibbald& Roberts 1998; Woods et al. 1989). Tobacco smoking is a chronic, relapsing disease (Fiore et al. 2008). Smoking cessation medications can temporarily help smokers become abstinent, but when the medication is stopped, smokers are able to return to baseline smoking during a washout period (Perkins et al. 2008, 2010a). During quit attempts, relapse is common even while still on medication (Fiore et al. 2008).

However, there are some issues that could preclude using a crossover design. One obvious concern is the possibility of carryover effects of the drug itself or any drug-induced changes in the outcome measures (Sibbald& Roberts 1998; Woods et al. 1989). The plasma half-life of gemfibrozil is 1.5 hours, so systemic concentration declines rapidly. There is no evidence, to our knowledge, that gemfibrozil causes permanent or long-lasting changes in neuronal or physiological systems. As stated above, any changes in smoking behavior due to the medication can be reversed when participants resume to ad libitum smoking in the crossover phase. In this study, participants will be told that they do not have to resume smoking if they achieve abstinence in the first phase, but they will not be able to continue in the study. To test for carryover effects, data will be analyzed for order of treatment condition.

Another potential threat to crossover designs is that participants will not remain blind to drug condition, which could bias their responses to the medication. Gemfibrozil is not psychoactive and has few side effects that distinguish it from placebo (see below). Even if participants are able to detect active drug versus placebo, results are not necessarily invalid. For example, Perkins et al. (2008, 2010a) reported that participants were not completely blinded to nicotine patch or varenicline. However, because correct identification of treatment condition did not differ according to quit interest of participants or reinforcement condition, Perkins et al. concluded that the observed therapeutic effect was not due to expectancy of benefit from the active medication.

Summary

The proposed study will be the first clinical evaluation of gemfibrozil as a novel medication for the treatment of tobacco dependence. We will use the screening methodology developed by Perkins et al. (2006, 2008, 2010a) to investigate the effect of gemfibrozil on smoking abstinence and on laboratory measures of nicotine reinforcement and cue-elicited craving. We will also examine the predictive relationship between these laboratory measures and smoking abstinence.

**Study Aims and Hypotheses**

The study aims are:

1. to investigate the effect of gemfibrozil on laboratory measures of nicotine reinforcement and cue-elicited craving
2. to screen for the ability of gemfibrozil to aid smoking abstinence during a brief quit attempt
3. to examine the validity of using laboratory measures of tobacco dependence to predict smoking abstinence and possible gemfibrozil-related increases in smoking abstinence.

Based on preclinical data discussed above, we hypothesize that:

1. Gemfibrozil will result in diminished nicotine reinforcement compared with placebo. This will be evidenced by participants choosing a lower percentage of puffs from nicotine cigarettes (vs. denic cigarettes) when taking gemfibrozil compared with placebo.
2. Gemfibrozil will result in an attenuated response to smoking cues. This will be evidenced by diminished autonomic responses (e.g., heart rate) and self-report responses (e.g., craving) to smoking cues (vs. neutral cues) when taking gemfibrozil compared with placebo.
3. Gemfibrozil will increase smoking abstinence. This will be evidenced by a greater number of days abstinent when taking gemfibrozil compared with placebo.
4. Laboratory measures of smoking will prove valid in predicting abstinence. This will be evidenced by percentage of puffs from nicotine cigarettes chosen in the forced-choice task and response to smoking cues being negatively associated with number of days abstinent.
5. Likewise, the difference in laboratory measures, when comparing the gemfibrozil and placebo conditions, will predict the difference in abstinence days, when comparing the gemfibrozil and placebo conditions.

**Participants**

This study will enroll up to 40 adult smokers who intend to quit smoking in the next 3 months. An eligibility checklist (Appendix 1) will be used during screening for admission to the study.

Inclusion Criteria

1. 19-65 year old males and females
2. smoking at least 10 cigarettes per day for at least 2 years
3. intend to quit smoking within the next 3 months
4. medically and psychologically healthy as determined by screening criteria (Appendix 2)
5. women capable of becoming pregnant must agree to use contraceptives during the study

Exclusion Criteria

1. currently attempting to quit smoking
2. treatment for tobacco addiction in the past 3 months
3. use of nicotine replacement products, bupropion, or varenicline in the past 3 months as an aid to quit or reduce smoking
4. use of any oral tobacco product in the past 3 months
5. history of drug or alcohol dependence within last 5 years
6. consumption of more than 15 alcoholic drinks per week on average during the past month
7. use of any illicit drug more than once per week on average during the past month
8. current use of gemfibrozil or other fibrate medication
9. current use of any medication that is contraindicated for gemfibrozil or that would interfere with the protocol in the opinion of the Qualified Investigator (QI).This includes, but is not limited to anticoagulants; statins; other fibrates; other lipid-lowering agents, such as colestipol, niacin, or herbal remedies; and any oral or injected medications for diabetes, such as rapaglinide.
10. any pre-existing gall-bladder disease or operation in the past 12 months
11. any history of or current cardiovascular, liver, hepatic or renal disease
12. diabetes
13. pregnant, nursing, or become pregnant during the study
14. Use of psychoactive drugs or medications as revealed by urine toxicology

**Study Design and Methods**

Overview

This outpatient study will be conducted at the Centre for Addiction and Mental Health (CAMH) under the supervision of Dr. Bernard Le Foll, Principal Investigator (PI). Le Foll received a Global Research Award for Nicotine Dependence from Pfizer, Inc. to conduct this study.

Recruitment

Participants will be recruited through posters, newspaper and magazine ads, CAMH research registry and web-based advertisements such as Craigslist, Kijiji and CAMH website. These methods have been used in the past as recruitment tools.

Cigarettes

For the forced-choice procedure, we will use Quest® 3 (Vector Tobacco Inc.) and commercially-available cigarettes. Quest® 3 cigarettes are manufactured using genetically engineered tobacco, such that a cigarette delivers less than 0.05 mg nicotine (Denic). The forced-choice procedure was validated in previous studies using these Quest® 3 cigarettes. A nicotine-containing cigarette (Nic) will be used for the other condition. For the cue-reactivity procedure, participants’ preferred brand of cigarette will be used.

Study Design

This study will use a double-blind, placebo-controlled, crossover design to investigate the effect of gemfibrozil on smoking abstinence and laboratory measures of tobacco addiction. Appendix 3 presents an overview of the study design. The study comprises two 2-week phases separated by a washout period of at least 1 week. Participants will receive gemfibrozil or placebo during the 2 week phases and will be assessed on laboratory measures at the end of the first week. The second medication week will serve as the quit attempt week. The second 2-week period will begin at least one week after the first, to allow for drug washout. Order of medication phases will be randomized and counterbalanced, such that half of the participants will receive gemfibrozil first followed by placebo, and the other half will receive placebo first followed by gemfibrozil. Participants will be contacted by telephone 1 week after the last medication phase, after which they will be discharged from the study. The Pharmacy will randomize the order of treatment of placebo or gemfibrozil.

Study Procedures

*Gemfibrozil/placebo dosing.* Lopid® (gemfibrozil, USP) tablets, each containing 600 mg gemfibrozil, will be supplied by Pfizer, Inc. CAMH Research Pharmacy will formulate the tablets into capsules and dispense to participants in blister packs. Gemfibrozil will be dosed as clinically indicated, 600 mg twice daily and orally, taken 30 minutes before the morning and evening meals. The participants will take 1 capsule of 600 mg at a time. Placebo capsules containing only lactose will follow the same regimen. A medication instruction form will be given to participants (Appendix 4)

*Medication compliance*. Medication compliance will be monitored by requiring participants to call into a dedicated phone line once a day when they have taken their second capsule; at this time they will also indicate the number of cigarettes they have smoked that day. Medication compliance will also be verified by requiring participants to return the blister packs, allowing staff to conduct a pill count verifying the number of doses taken. Finally, as described below, a blood sample will be taken in order to verify the levels of gemfibrozil in the body.

*Blood samples*. During the initial visits where eligibility for inclusion in the study will be assessed, a blood sample will be drawn to assess measures including serum glucose, serum creatinine, creatine kinase, complete blood counts, liver function tests, PT/INR and HbA1c as outlined in Appendix 2.

Two samples of blood will be drawn after at least 10 days on medication and during each phase. One blood sample will be processed for subsequent quantitative analysis of gemfibrozil concentration. Blood samples will be stored in a secure -80 freezer. A few placebo samples will be sent to the laboratory for testing to insure the specificity of the assay. Plasma samples will be destroyed after analyses are completed. Additionally, the other blood sample will be drawn for medical safety monitoring (CBC, BUN/Cr, CK, PT/INR, HbA1c, and LFTs) to monitor any hematologic changes or change in renal/liver function.

*Genetic Blood Samples.* At the start of the study, a 20-ml blood sample will be obtained by venipuncture for genotyping. Examples of the genes of interest include, but are not limited to dopaminergic (DRD1-5), dopamine metabolic (COMT), nicotine metabolic (CYP2A6), nicotinic (CHRNA4-5, CHRNB2), and cannabinoid (CB1R) genes, all of which have been associated with tobacco addiction. Blood samples will be frozen and stored in a secure freezer used by the Translational Addiction Research Laboratory. Identifiers, such as name or date of birth, are not included with these samples.  Genetic data will be stored on a password protected computer in a locked room. The data files are also password protected.   Samples will be stored indefinitely as additional polymorphisms relevant to addiction may develop.  Exploratory genotyping may be undertaken to assess the role of other genes that future research suggests to be relevant to the purpose of this protocol. For example, a gene or a newly defined polymorphism may be suggested to affect processes related to nicotine reinforcement or cue-elicited tobacco craving. Any use of stored samples for future genetic testing will be brought to the REB for approval.

*Assessment visit.* Upon attending the clinical trial site at CAMH, the participant will first be requested to provide written informed consent. After obtaining consent, participants will be asked to provide general demographic information and detailed questions regarding their tobacco and other drug use (Appendix 5; Appendix 17). Participants will also be requested to undergo a psychiatric assessment (MINI; Beck Depression Inventory) and assessment of usage of psychiatric medications (concomitant medication; medication history). A complete medical history and physical exam will be conducted to assess general health and to assess screening criteria (e.g. adverse events and Appendix 13 at baseline). A blood and urine sample will also be taken to assess measures as outlined in Appendices 1 and 2.

*Medication collection visit.* During the first clinic visit of each 2-week phase, participants will collect their medication and complete a number of baseline questionnaires. During this visit, all participants will provide a breath carbon monoxide (CO) sample, report the number of cigarettes smoked over the past 24 hours, and complete the following baseline questionnaires:

1. Fagerström Test for Nicotine Dependence (FTND; Heatherton et al. 1991; Appendix 6)
2. Mood Form (Diener& Emmons 1984; Appendix 7)
3. Tobacco Craving Questionnaire-Short Form (TCQ-SF; Heishman et al. 2008; Appendix 8)
4. Visual Analog Scale (VAS) items assessing mood and craving for a cigarette (Appendix 9)
5. Minnesota Nicotine Withdrawal Scale (MNWS; Hughes and Hatsukami 1998; Appendix 10)
6. Smoking Contemplation Ladder (Rustin and Tate 1993; Appendix 11)
7. Timeline follow back (Appendix 17)

Participants will be given the medication instruction form (Appendix 4) and daily call material (Appendix 15).

*Experimental sessions*. During the next clinic visit, one week after starting the medication, participants will undergo two laboratory sessions, one cue-reactivity and one forced choice. The cue-reactivity and forced choice may be conducted on separate days if required by conflicting schedules.

a) *Cue-reactivity.* The cue-reactivity session occurs after one week of medication. There are two cue conditions tested in the session, one with smoking cues and the other with nonsmoking cues. Order of the two conditions will be counterbalanced across participants. Participants start by completing baseline self-report measures (TCQ-SF, Mood Form, VAS, MNWS). Cue conditions are then begun. Each cue condition begins with participants seated in a comfortable chair and smoking 4 puffs from their preferred-brand cigarette. Participants are then presented with a tray containing an opaque cover. In the smoking-cue condition, a pack of the participant’s preferred brand of cigarettes, a lighter, and an ashtray are under the tray cover. In the nonsmoking-cue condition, a pack of unsharpened pencils, a pencil sharpener, and a small notepad are under the cover. Participants are then connected to physiological recording devices to measure skin conductance, skin temperature, heart rate and blood pressure. This is followed by 10 minutes of audio-instructed progressive relaxation. Participants are then given the Mood form, TCQ-SF and VAS. After resting for 5 minutes, baseline physiological measures are recorded for 5 minutes. When instructed, the participant lifts the cover on the tray. In the smoking-cue condition, participants take one cigarette out of the pack, light it without puffing, hold it for 1 minute, extinguish the cigarette, and replace the cover on the tray. In the nonsmoking-cue condition, participants take one pencil out of the pack, sharpen it, hold it for 1 minute as if to write on the notepad, and then replace the cover. Participants complete the TCQ-SF, Mood Form, and VAS immediately and 15 minutes after cue presentation; physiological measures are recorded continuously.

*b) Forced-choice.* The forced-choice session occurs later that same afternoon. After a brief lunch period, participants will smoke 4 puffs from their preferred-brand cigarette and then relax (read or listen to music) for 30 minutes, during which they are not allowed to smoke. They then complete baseline self-report measures (TCQ-SF, Mood Form, VAS) and give a breath CO sample. The forced-choice session consists of exposure trials and choice trials. During exposure trials, participants sample the Nic and Denic cigarettes. Exposure order will be counterbalanced across subjects, and each cigarette will be sampled twice for a total of 4 trials, using either an ABAB or BABA order. At 30-minute intervals, participants smoke 4 puffs from the designated cigarette. After each exposure trial, participants rate the puffs using the modified Cigarette Evaluation Questionnaire (mCEQ; Cappelleri et al. 2007; Appendix 12). The choice trials begin 30 minutes after the last exposure trial. There are 4 forced-choice trials occurring at 30-minute intervals; participants choose any combination of 4 puffs from the two cigarettes at each trial. During intervals between exposure and choice trials, participants can read or listen to music. The smoking rate during the entire session (8 puffs every 60 minutes) is less than that of the typical ad libitum smoking pattern of a daily smoker of one cigarette (10 puffs) every 30-40 minutes (Hatsukami et al. 1988). At the end of the session, participants give a breath CO sample and complete the TCQ-SF, Mood Form, and VAS.

*Abstinence visit*. Participants will then visit the clinic again one week after the laboratory session to assess abstinence following the 1 week quit attempt while on medication. In this visit, they will be administered a number of questionnaires, their CO measures taken and questions about adverse events will be asked.

During the medication phase of the study, participants will call the clinic each day to report that they have taken their medication and the number of cigarettes that they have smoked that day.

At each clinic visit during the study, participants will complete the Mood Form, TCQ-SF, MNWS, and provide a breath CO sample. Participants will be asked whether they think they are taking gemfibrozil or placebo. Additionally, participants will report whether they smoked over the past 24 hours. Breath CO < 5 ppm will be used to verify smoking abstinence (Marrone et al. 2011). Concomitant medication, adverse events and side effects (Appendix 13) will be assessed at each clinic visit.

Women must provide a negative urine pregnancy test at each clinic visit and must not be nursing because the effects of gemfibrozil on fetal and early development are not known.

During the study, participants will consume their normal caffeine intake and continue to take prescription medications, unless otherwise instructed by the Qualified Investigator. Participants will smoke normally and must not use any illicit drugs or alcohol 24 hours before experimental sessions. To ensure that participants are not under the influence of recent alcohol or drug consumption, their blood alcohol concentration (BAC) will be tested with a breathalyzer (Heishman et al., 1996, 1998). Participants must register a BAC of 0.000%. If participants fail the BAC, their session will be canceled, and they will not receive payment that day.

End of Participation and Follow Up

At the conclusion of the study, either the PI or his designee will meet with participants to answer any questions they might have about the study. This also serves as an opportunity to intervene with respect to the participant’s tobacco dependence. The investigator will counsel participants on the risks of continued smoking and the benefits of quitting and give them a referral list of smoking treatment programs.

We will call participants 1 week after the last medication phase to assess potential side effects (see Appendix 13) and their general health. The QI will review the follow-up assessment, and if there are no problems, the participant will be discharged from the study. Any medical concerns will be followed until a satisfactory resolution is reached.

Figure 1 provides a schematic of the study design

Appendix 3 provides a schematic of the trial design

**Risks and Discomforts**

Minimal risks/discomforts

There is a minor risk of contact dermatitis as a result of the skin electrodes. Participants will likely experience increased tobacco craving after presentation of smoking cues. Because both Nic and Denic cigarettes are smoked during the forced-choice session, participants will not experience withdrawal. Smoking the equivalent of 4 cigarettes over 4 hours during the forced-choice session should pose little additional risk to current smokers. Because smoking is toxic to fetal development and because the effects of gemfibrozil on fetal development are not known, women must provide a negative urine pregnancy test before sessions and must not be nursing.

There is a possibility of discomfort at the site of needle entry for blood draws, and there is a risk of bruising. There is a remote risk of fainting or local infection. Trained phlebotomists and nurses using aseptic techniques will draw blood.

Participants might experience nicotine withdrawal during the quit-attempt weeks. This might include cigarette cravings, depressed mood, irritability, insomnia, anxiety, restlessness, difficulty concentrating, and increased appetite (DSM-IV symptoms of nicotine withdrawal, American Psychiatric Association 2000). These symptoms might increase the possibility of relapse to smoking.

Potential loss of confidentiality is a particular risk of studies involving genotyping. Under such circumstances, it could be a risk for genetic information about an individual to be made known. For example, genetic information could be informative to determine parentage or could be interpreted by an insurance company or employer to mean that the person was at increased risk for a problem. No genotype data will be released to subjects under any circumstance. In the study, there will be no identification of genetic diseases, and thus, no relevant information to report to subjects.

More than minimal risks/discomforts

The primary risks of this study involve gemfibrozil administration. In the Helsinki Heart Study, 2046 patients received gemfibrozil for up to 5 years. The adverse reactions that were statistically more frequent in the drug group compared with the placebo group were gastrointestinal reactions (34% vs. 24%), including dyspepsia and abdominal pain. Acute appendicitis (1.2% vs. 0.6%) and

Figure 1

atrial fibrillation (0.7% vs. 0.1%) were documented less frequently. From other clinical studies, there is a probable causal relation between gemfibrozil and the following: viral and bacterial infections (common cold, cough, urinary tract infection), dizziness, somnolence, paresthesia,

peripheral neuritis, decreased libido, depression, headache, blurred vision, impotence, musculoskeletal pain, muscle spasms, increased liver transaminases, anemia, angio- and

laryngeal edema, increased malignancies, bleeding, hypoglycemia, gall bladder disease, and rash. These reactions or illnesses occurred in patients who took gemfibrozil for several years (see appended package label). There are also possible adverse interactions between gemfibrozil and certain medications, including HMG-CoA reductase inhibitors (e.g., statins), anticoagulants (e.g., heparin), blood glucose-lowering drugs (e.g., rapaglinide), and bile acid-binding resins (e.g., colestipol). In this protocol, the likelihood of serious adverse effects from gemfibrozil in healthy volunteers taking the medication for 2 weeks is low; however, participants will be closely monitored for side effects.

**Participant Safety Monitoring**

During screening, study applicants will undergo a complete battery of medical laboratory tests and physical exam to determine their eligibility and safety of their participation in this study (see Appendix 2). Study applicants will be excluded if they have any history of liver disease, abnormal renal function, diabetes, recent gallbladder disease or operation, or any other medical condition, or are on any medication that may contraindicate their participation in this study. During the treatment phase of the study, participants will be asked about side effects and adverse events at each clinic visit (Appendix 13). Additionally, CBC, complete metabolic panel, creatine kinase levels, PT/INR, and HbA1c will be assessed at screening and after at least 10 days on medication. The QI/PI (Dr. Bernard Le Foll) will review side effect reports and medical tests. Participants will be discharged from the study if they have any signs or symptoms that may contraindicate continued drug administration.

If participants seek medical care during the study, they will be instructed to notify their physician or medical care provider of involvement in the study and the possibility that they may be taking gemfibrozil. We will follow up with participants 1 week after the last medication phase to assess potential side effects (see Appendix 13). The QI will review the follow-up assessment, and if there are no problems, the participant will be discharged from the study. Any medical concerns will be followed until a satisfactory resolution is reached.

Successful Quit Attempts: Ethical considerations

The crossover design creates a special case in which a participant may successfully maintain abstinence during the first quit attempt. Although participants always have the autonomy to discontinue study participation, this is one situation where it could benefit them to do so. Thus, participants who successfully maintain abstinence during the first phase will meet with the PI or their designee to discuss discontinuing their participation. Participants will be informed of the dangers of returning to smoking and the benefits of continued abstinence. Participants will be informed of the treatment opportunities that are available to them, but that they cannot continue on study medication. Referrals for further treatment, both pharmacological and behavioral, will be provided. Participants will be encouraged to drop out of the study and continue smoking abstinence, but will not be arbitrarily discharged from the study. This procedure strikes a balance between two fundamental ethical principles of clinical research, beneficence and respect for the autonomy of the participant.

Withdrawal of subjects:

Stop points for withdrawing participants from the study include: 1) at the request of the participant, 2) if the participant is unable or unwilling to comply with study requirements and procedures, 3) any occurrence that in the opinion of the investigators would compromise the health of the participant, 4) any serious adverse effect; and 5) a successful quit attempt after the first phase of the study, meaning that the participant does not complete the second phase of the study.

When a subject is withdrawn from a study, data collection will cease with the exception of monitoring for adverse events and follow-up to check up on health, especially in the event where the participants withdrew for health reasons. Subjects will be replaced in the sense that attempts will be made to meet the target enrollment and continue to recruit until this has been met.

**Outcome measures**

Primary outcome measures include laboratory assessments of nicotine reinforcement and smoking cue reactivity. The measure of nicotine reinforcement is the percentage of nicotine cigarette puffs chosen during the forced-choice task. Measures of cue reactivity include tobacco craving, mood, and autonomic responsivity.

The secondary outcome measure is days of smoking abstinence during the quit-attempt weeks. Abstinence is assessed by self-reports of no smoking and by breath carbon monoxide < 5 ppm. Other assessments of abstinence include self-reported tobacco craving and withdrawal.

Sociodemographic and baseline measures (e.g., smoking history, level of nicotine dependence, number of cigarettes smoked per day, tobacco craving, mood, smoking motivations) will be used to characterize the sample and explore as mediators of primary and secondary outcomes.

**Statistical Analysis**

Standard techniques will be used to examine the distributional properties of the data (means, standard deviations, medians, skewness, kertosis) in order to verify that all assumptions are met for subsequent analyses. The data will be examined for univariate and multivariate outliers. For all analyses, results are considered significant at *p*<.05.

Aim 1:Investigate the effect of gemfibrozil on laboratory measures of nicotine reinforcement and cue-elicited craving

The percentage of puffs chosen from Nic cigarettes in the forced-choice sessions will be analyzed using mixed-model analysis of variance (ANOVA) with drug condition (placebo, gemfibrozil) as within-subject factors and order of exposure of drug condition and order of exposure of cigarette type (Nic, Denic) as between-subject factors. For cue-reactivity sessions, self-report and physiological measures will be analyzed by mixed-model ANOVA, with drug condition (placebo, gemfibrozil), cue type (smoking, nonsmoking) and time post-cue (0, 15 minutes) as within-subject factors and order of exposure of drug condition and cue type as between-subject factors. For measures showing significant main effects or interactions, post hoc comparisons between means will be conducted. Supplemental analyses may be conducted using bivariate analyses to identify relationships to participant-level variables (e.g., socioeconomic factors, mood). Variables identified will be used as covariates if they are believed to be suppressor variables or entered into stepwise regressions to better characterize the participants for whom gemfibrozil has the greatest effect on laboratory measures.

Aim 2: Screen for the ability of gemfibrozil to aid smoking abstinence during a brief quit attempt

Data from the quit-attempt will be analyzed using mixed-model ANOVA with drug condition (placebo, gemfibrozil) as a within-subject factor and order of exposure of drug condition as a between-subject factor. Post hoc comparisons between means will be conducted on significant overall effects. Supplemental analyses may be conducted using bivariate analyses to identify relationships to participant-level variables (e.g., socioeconomic factors, mood). Variables identified will be used as covariates if they are believed to be suppressor variables or entered into stepwise regressions to better characterize the participants for whom gemfibrozil facilitates smoking abstinence.

Aim 3: Examine the validity of using laboratory measures of tobacco dependence to predict smoking abstinence and possible gemfibrozil-related increases in smoking abstinence

Bivariate correlational analyses will be used to determine the predictive association between laboratory measures of nicotine reinforcement and cue reactivity and tobacco abstinence during the quit-attempt. Two multivariate stepwise regressions will then be constructed to determine multivariate predictors of abstinence days and gemfibrozil-related change in abstinence days. At step one, demographic variables will be entered. At step two, smoking-related characteristics that are significantly associated with abstinence will be entered. Finally, at step three, measures of laboratory performance in both forced-choice and cue-reactivity that were significant in bivariate analyses will be entered to determine their multivariate predictive validity for smoking abstinence.

Power Analysis

Based on the preclinical data, we expect that gemfibrozil will decrease cue-reactivity, the number of puffs taken on the nic cigarette and increase abstinence. The preclinical data suggest that observed effect size of those effects will be around 0.5. However, those effect sizes may be lower in human subjects. As we have currently no data on human subjects, we decided to power the study to be able to detect a smaller effect size. Taken into account the variance of the behavioral responses based on the previous human laboratory studies (McKee et al., 2011), with 40 subjects, we will have sufficient power (>80%) at an alpha level of 0.05 to detect differences between conditions (gemfibrozil vs placebo) as small. This study will provide the necessary data to determine the effect size of gemfibrozil on these measures and preliminary evidence on the potential utility of gemfibrozil as a smoking cessation pharmacotherapy. Based on the results obtained, we can submit further grants to conduct a clinical trial to investigate the efficacy of gemfibrozil as a smoking cessation medication.

Missing, unused and spurious data:

Missing data will be dealt with according to statistical principles for filling in missing cells. In the event that large amounts of data are missing due to, for example, participant noncompliance with study procedures, the data will be analysed according to the primary goals of the study and the objectives of this study. Statistical procedures that deviate from the protocol will be reported and justified in a protocol deviation log.

The data from all eligible subjects will be included in the data analysis

Accrual Number

There are several factors that could adversely affect accrual and completion of this study: a) participants may quit during the first medication phase; b) some participants might experience intolerable side effects of gemfibrozil; and c) some participants might not be compliant with taking medication. Given these factors, we anticipate up to 50% attrition. Drop-outs will be replaced.

**Human Subjects Protections**

Equity of Subject Selection

Participants will be enrolled without regard to sex, race, or ethnicity. Efforts will be made to include ethnic minorities in proportion to their presence in the metropolitan Toronto area (http://www.toronto.ca/demographics/index.htm). Targeted enrollment figures for Toronto are shown below.

Targeted enrollment figures for Toronto are shown below.

| **APPROVED STUDY POPULATION** | | | | | | |
| --- | --- | --- | --- | --- | --- | --- |
|  | FEMALE  ENROLLMENT | MALE  ENROLLMENT | **TOTAL**  ENROLLMENT | FEMALE  COMPLETERS | MALE  COMPLETERS | **TOTAL**  COMPLETERS |
| **APPROVED**  **CEILING** | 20 | 20 | 40 | 15 | 15 | 30 |

| **TARGETED/PLANNED ENROLLMENT** | | | |
| --- | --- | --- | --- |
| **ETHNIC CATEGORY** | Sex/Gender | | |
|  | Females | Males | Total |
| Hispanic or Latino | 1 | 1 | 2 |
| Not Hispanic or Latino | 19 | 19 | 38 |
| **Ethnic Category: Total of All Subjects*** | 20 | 20 | 40 |
| **RACIAL CATEGORIES** |  |  |  |
| American Indian/Alaska Native | 0 | 0 | 0 |
| Asian | 1 | 1 | 2 |
| South Asian | 1 | 1 | 2 |
| Black or African American | 1 | 1 | 2 |
| White | 17 | 17 | 34 |
| **Racial Categories: Total of All Subjects*** | 20 | 20 | 40 |

Exclusion Justifications

Smokers indicating no plans to quit smoking in the next 3 months, receiving smoking cessation treatment, or using any medications to help them quit smoking will be excluded because the study requires participants to make a quit attempt. Heavy drug and alcohol use could potentially confound responses to smoking cues and reinforcing effects of cigarettes. Children will be excluded because smoking under age 19 is illegal, and it is unethical to encourage teenage smoking. Pregnancy and nursing are exclusionary because smoking is toxic to fetal and infant development and the effect of gemfibrozil on pregnancy and nursing mothers is unknown.

Qualifications of Investigators and Study Staff

Bernard Le Foll, M.D., Ph.D. is Head of the Translational Addiction Research Laboratory at the Center for Addiction and Mental Health (CAMH) in Toronto. He has over 15 years of experience in conducting animal and human research on the determinants of nicotine addiction. He will serve as PI and QI at CAMH and will be responsible for enrolling and testing participants on the same protocol. Dr. Le Foll will be responsible for analysis and interpretation of data and in writing of abstracts and manuscripts. Dr. Le Foll will also screen participants for inclusion/exclusion criteria and monitor adverse events in Toronto.

Patricia Di Ciano, Ph.D. is a postdoctoral fellow in the Translational Addiction Research Laboratory. She graduated in 1999 with a doctorate in biopsychology from The University of British Columbia in Vancouver. Dr. Di Ciano has worked on the neurobiological basis of addiction. Dr. Di Ciano will function as lead AI and will obtain informed consent from participants, assist with the conduct of experimental sessions, monitor data accuracy, oversee data entry into spreadsheets, participate in report writing and other tasks as needed.

**Benefits**

This study does not offer direct benefit to participants. However, it will be the first clinical evaluation of gemfibrozil on nicotine reinforcement and cue-elicited tobacco craving. Additionally, the study will yield generalizable knowledge about the effects of gemfibrozil as a novel medication for the treatment of tobacco dependence.

**Classification of Risk**

Participation in this study represents more than minimal risk and offers no direct benefit to participants. The risks are reasonable in relation to the anticipated benefit of knowledge regarding a novel medication for the treatment of tobacco dependence.

**Consent Documents and Process**

Participants who meet the eligibility criteria will give written informed consent. Drs Le Foll or Di Ciano will obtain informed consent at CAMH. They are experienced researchers and are knowledgeable about the pharmacology and side effect profile of gemfibrozil. Thus, they are qualified to answer questions about the study’s procedures and risks associated with gemfibrozil. Other individuals may collect informed consent if they meet all the requirements and have sufficient training experience and education.

The consent form contains all required elements. Excluding the required boilerplate sections, the Flesch-Kincaid reading level of the consent form is 8.0. Participants will be encouraged to ask questions about anything they don’t understand as the consent is being read. After all questions have been answered to the participant’s satisfaction, they will complete a 10-question true/false quiz (Appendix 14) to test their understanding of the procedures and risks. A minimum score of 80% correct answers after two trials is required for participation.

**Data and Safety Monitoring**

This study will use a double-blind, placebo-controlled, crossover design to investigate the effect of gemfibrozil versus placebo on smoking abstinence and laboratory measures of tobacco addiction. The sample will be 40 healthy smokers between the ages of 19 and 65 years of age who intend to quit smoking in the next 3 months.

Data Monitoring Plan

Data will be collected using computerized or standardized paper forms and will only be identified with the participant’s study ID number. The code that links the name of the participant and the study ID will be kept confidential by the PI in a secured computer within a locked office. Paper-form data will be entered in a computer by research staff, and checked by independent staff.

Safety Monitoring Plan

The occurrence of AEs will be assessed at baseline and each clinic visit during the medication phase of the study. Study investigators will follow all AEs to the point of a satisfactory resolution. All AEs will be assessed to determine if they meet criteria for a serious adverse event (SAE). As defined by Health Canada, SAEs will be systematically evaluated at each clinic visit. Any SAE, whether or not related to study medication, will be reported to the CAMH REB and Pfizer. If this SAE meets requirements for reporting to Health Canada, this information will be submitted according to reporting requirements and time frames.

If a participant withdraws from the study or if an investigator discharges a participant because of an SAE, the participant will have appropriate follow-up medical monitoring. Monitoring will continue until the problem requiring hospitalization has resolved or stabilized with no further change expected, is clearly unrelated to study medication, or results in death.

**Blinding and Unblinding**

This will be a double-blind experiment with respect to whether the participant is receiving gemfibrozil or placebo. The Pharmacy will assign the participants to order of treatment in this crossover design.

For unblinding during normal business hours, the QI will contact the Research Pharmacy who will provide the information.

Unblinding may also occur at the request of the participant after withdrawal from the study. This will occur during normal business hours.

In the case of a medical emergency, unblinding will proceed according to the Translational Addiction Research Laboratory SOP entitled: ‘Clinical Research Emergencies and Unblinding Procedures’, version 1.0 (December 12, 2012). The procedure is outlined below and may be revised according to the SOP.

In the case of a medical emergency, the QI will determine whether unblinding is necessary to ensure appropriate medical care is provided. To do so, the QI will notify the Nursing Supervisor/Charge Nurse on duty through hospital locating and inform them of the need for unblinding a research study. The Nursing Supervisor will make preparations to open the night cupboard which houses randomization codes. The QI will discuss with the Nursing Supervisor the appropriate steps required to obtain this information. This can entail the Nursing Supervisor obtaining this information from the Inpatient Pharmacy in the College St. site or marking appropriate preparations to allow the QI to obtain this information directly.

After unblinding and management, the QI will document the circumstances necessitating unblinding and the fact that unblinding took place for this participant. While not required, a notice should be sent to pharmacy informing them of unblinding should they require this information for the Investigational Product logs, etc.

**Quality Assurance**

All monitoring will be conducted in accordance with CAMH regulations for Quality Assurance.

**Adverse Event Reporting**

Serious adverse events, adverse events, and unanticipated problems will be reported according to CAMH guidelines.

**Alternatives to Participation**

Participants do not receive any treatment in this study. The alternative, therefore, is not to participate.

**Confidentiality.** Confidentiality of participants will be maintained at all times. No identifiable information will be released to outside parties without the written permission of the participant. All forms with information about participants will be coded with a unique study number. Data collected will be stored in computerized spreadsheets and will be identified only by the participant’s study number. All forms and data will be kept in locked files. Only study investigators will have access to the data.

Potential loss of confidentiality is a particular risk of studies involving genotyping. As a result, a number of additional safeguards against transmission of this information to unauthorized individuals have been established. Subject ID numbers and not subject names will accompany blood samples. The data will be rigorously protected as per standard procedures, i.e. double locked, no access except to authorized study personnel, separate locations for ID codes and subject names, etc. No genotype data will be released to subjects under any circumstance. No CLIA- (Clinical Laboratory Improvement Act) certified genotyping acceptable for a clinical diagnostic or insurance purpose will be performed. In the study, there will be no identification of genetic diseases, and thus, no relevant information to report to subjects.

**Conflict of Interest**

There are no conflicts to report. Dr. Le Foll (CAMH) will serve as overall study PI and will receive the grant money from Pfizer. Pfizer manufactures gemfibrozil and will provide study medication free of charge. Investigators will provide Pfizer with a written report at the conclusion of the study, which will not contain personal identifiers of participants.

**Compensation**

Participants will be compensated for their time and inconvenience while in the study and will be paid at each visit. Compensation for the phone calls will be provided at the end of the study. If participation is stopped for medical or scientific reasons, participants will receive compensation for the amount of the study completed to that point, including completion incentives. If participants voluntarily withdraw from the study, they will be compensated for the amount of participation to the point of withdrawal, but will not receive a completion incentive. Approximate compensation is as follows.

CAMH

- Assessment visit - $30
- Completion of first laboratory visit - $150
- Completion of abstinence week - $150
- Completion of washout period - $60
- Completion of second laboratory visit $150
- Completion of abstinence week - $150
- Completion of entire study - $90
- Completing the phone calls - $2 per day

Total compensation, not including compensation for phone calls: $780

This trial will be conducted in compliance with the protocol, GCP and the applicable regulatory requirements.

**Criteria for the termination of the trial:**

The trial will be terminated if, in the opinion of the QI, the health and/or safety of the participants is compromised

The investigator/institution will permit trial-related monitoring, audits, REB review, and regulatory inspections(s), providing direct access to source data/documents.

**Data Handling and Record Keeping:**

Data will be entered directly into computer programs on to paper and will then be transferred to Case Report Forms. Source documents will be kept in a locked cabinet with identifying information removed. Source documents will be reviewed by the QI on a regular basis. All data will be archived, as required, for 25 years.

**Investigational Product Accountability**

Appendix 16 provides a statement from the CAMH Pharmacy regarding investigational product accountability**References**

Abrams DB, Monti PM, Carey KB, Pinto RP, Jacobus SI (1988) Reactivity to smoking cues and relapse: two studies of discriminant validity. Behav Res Ther 26:225-233.

American Psychiatric Association (2000) Diagnostic and statistical manual of mental disorders, 4^th^edn text revision. American Psychiatric Association, Washington, DC

Cappelleri JC, Bushmakin AG, Baker CL, Merikle E, Olufade AO, Gilbert DG (2009) Confirmatory factor analyses and reliability of the modified cigarette evaluation questionnaire. Addict Behav 32:912-923.

Carter BL, Tiffany ST (1999) Meta-analysis of cue-reactivity in addiction research.Addiction94:327-340.

Centers for Disease Control (2009) Cigarette smoking among adults and trends in smoking cessation-United States, 2008.Morbidity and Mortality Weekly Report 58:1227-1232.

Corrigall WA (1999) Nicotine self-administration in animals as a dependence model.Nicotine Tob Res1:11-20.

de Wit H, Johanson CE (1987) A drug preference procedure for use with human volunteers. In Bozarth MA (ed) *Methods of Assessing the Reinforcing Properties of Abused Drugs*, pp. 559-572. New York: Springer-Verlag.

Diener E, Emmons RA (1984) The independence of positive and negative affect. J PersonalSocPsychol47:1105-1117.

Drobes DJ, Tiffany ST (1997) Induction of smoking urge through imaginal and *in vivo* procedures: physiological and self-report manifestations. J AbnPsychol106:15-25.

Fegley D, Gaetani S, Duranti A et al. (2005) Characterization of the fatty acid amide hydrolase inhibitor cyclohexylcarbamic acid 3'-carbamoyl-biphenyl-3-yl ester (URB597): effects on anandamide and oleoylethanolamide deactivation. J PharmacolExpTher 313:352-358.

Fiore MC, Jaén CR, Baker TB et al (2008) Treating tobacco use and dependence: 2008 update. U.S. Department of Health and Human Services, Rockville, MD.

Fruchart JC, Duriez P, Staels B (1999) Peroxisome proliferator-activated receptor-alpha activators regulate genes governing lipoprotein metabolism, vascular inflammation and atherosclerosis. CurrOpinLipidology 10:245-257.

Gonzalez S, Cascio MG, Fernandez-Ruiz J, Fezza F, Di Marzo V, Ramos JA (2002) Changes in endocannabinoid contents in the brain of rats chronically exposed to nicotine, ethanol or cocaine. Brain Res 954:73-81.

Griffiths RR, Bigelow GE, Henningfield JE (1980) Similarities in animal and human drug-taking behavior. In Mello NK (ed) *Advances in Substance Abuse, vol 1*, pp. 1-90. Greenwich, CT: JAI Press.

Harvey DM, Yasar S, Heishman SJ, Panlilio LV, Henningfield JE, Goldberg SR (2004) Nicotine serves as an effective reinforcer of intravenous drug-taking behavior in human cigarette smokers. Psychopharmacology 175:134-142.

Hatsukami DK, Pickens RW, Svikis DS, Hughes JR (1988) Smoking topography and nicotine blood levels. Addict Behav 13:91-95.

Heatherton TF, Kozlowski LT, Frecker RC, Fagerström KO (1991) The Fagerström test for nicotine dependence: A revision of the Fagerström Tolerance Questionnaire. Br J Addiction86:1119-1127.

Heishman SJ, Singleton EG, Crouch DJ (1996) Laboratory validation study of Drug Evaluation and Classification program: Ethanol, cocaine, and marijuana. J Anal Toxicol20:468-483.

Heishman SJ, Singleton EG, Crouch DJ (1998) Laboratory validation study of Drug Evaluation and Classification program: Alprazolam, *d*-amphetamine, codeine, and marijuana. J Anal Toxicol22:503-514.

Heishman SJ, Saha S, Singleton EG (2004) Imagery-induced tobacco craving: duration and lack of assessment reactivity bias. Psychol Addict Behav 18:284-288.

Heishman SJ, Boas ZP, Hager MC, Taylor RC, Singleton EG, Moolchan ET (2006) Effect of tobacco craving cues on memory encoding and retrieval in smokers. Addict Behav 31:1116-1121.

Heishman SJ, Singleton EG, Pickworth WB (2008) Reliability and validity of a short form of the Tobacco Craving Questionnaire. Nicotine Tob Res 10:643-651.

Heishman SJ, Lee DC, Taylor RC, Singleton EG (2010) Prolonged duration of craving, mood, and autonomic responses elicited by cues and imagery in smokers: effects of tobacco deprivation and sex. ExpClinPsychopharmacol 18:245-256.

Hughes J, Hatsukami DK (1998) Errors in using the tobacco withdrawal scale. Tob Control 7:92-93.

Keating GM (2011) Fenofibrate: a review of its lipid-modifying effects in dyslipidemia and its vascular effects in Type 2 dibetes mellitus. Am J Cardiovasc Drugs 11:227-247.

Koob GF (2008) New dimensions in human laboratory models of addiction. Addict Biol 14:1-8.

Lee DC, Myers CS, Taylor RC, Moolchan ET, Heishman SJ (2007) Consistency and reliability of subjective responses to imagery-induced tobacco craving over multiple experimental sessions. Addict Behav 32:2130-2139.

Lerman C, LeSage MG, Perkins KA, O’Malley SS, Siegel SJ, Benowitz NL, Corrigall WA (2007) Translational research in medication development for nicotine dependence.Nature Rev 6:746-762.

Marrone GF, Shakleya DM, Scheidweiler KB, Singleton EG, Huestis MA, Heishman SJ (2011) Relative performance of common biochemical indicators in detecting cigarette smoking. Addiction 106:1325-1334.

Mascia P, Pistis M, Justinova Z et al. (2011) Blockade of nicotine reward and reinstatement by activation of alpha-type peroxisome proliferator-activated receptors. Biol Psychiatry 69:633-641.

McKee SA, Sinha R, Weinberger AH, et al. Stress decreases the ability to resist smoking and potentiates smoking intensity and reward*.* J Psychopharmacol 2011; 25:490-502.

Melis M, Pillolla G, Luchicchi A,[Muntoni AL](http://www.ncbi.nlm.nih.gov/pubmed?term=%22Muntoni%20AL%22%5BAuthor%5D), [YYasar S](http://www.ncbi.nlm.nih.gov/pubmed?term=%22Yasar%20S%22%5BAuthor%5D), [Goldberg SR](http://www.ncbi.nlm.nih.gov/pubmed?term=%22Goldberg%20SR%22%5BAuthor%5D), [Pistis M](http://www.ncbi.nlm.nih.gov/pubmed?term=%22Pistis%20M%22%5BAuthor%5D)(2008)Endogenous fatty acid ethanolamides suppress nicotine-induced activation of mesolimbic dopamine neurons through nuclear receptors. J Neurosci 28:13985-13994.

Melis M, Carta S, Fattore L, Tolu S, Yasar S, Goldberg SR, Fratta W, Maskos U, Pistis M (2010) Peroxisome proliferator-activated receptors-alpha modulate dopamine cell activity through nicotinic receptors. Biol Psychiatry 68:256-264.

Niaura R, Abrams D, Demuth B, Pinto R, Monti P (1989) Responses to smoking-related stimuli and early relapse to smoking. Addict Behav 14:419-428.

Niaura R, Abrams DB, Pedraza M, Monti PM, Rohsenow DJ (1992) Smokers’ reactions to interpersonal interaction and presentation of smoking cues. Addict Behav17:557-566.

Panlilio LV, Justinova Z, Mascia P, Pistis M, Luchicchi A, Lecca S, Barnes C, Redhi GH, Adair J, Heishman SJ, Yasar S, Aliczki M, Haller J, Goldberg SR (2012) Novel use of a lipid-lowering fibrate medication to prevent nicotine reward and relapse: preclinical findings. Neuropsychopharmacology, advance online publication, doi:10.1038/npp.2012.31.

Perkins KA, Grobe JE, Weiss D, Fonte C, Caggiula A (1996) Nicotine preference in smokers as a function of smoking abstinence. PharmacolBiochemBehav 55:257-263.

Perkins KA, Stitzer, Lerman C (2006) Medication screening for smoking cessation: a proposal for new methodologies. Psychopharmacology 184:628-636.

Perkins KA, Lerman C, Stitzer ML, Fonte CA, Briski JL, Scott JA, Chengappa KNR (2008) Development of procedures for early screening of smoking cessation medication in humans. ClinPharmacolTher 84:216-221.

Perkins KA (2009) Does smoking cue-induced craving tell us anything important about nicotine dependence? Addiction 104:1610-1616.

Perkins KA, Lerman C, Fonte CA, Mercinavage M, Stitzer ML, Chengappa KNR, Jain A (2010a) Cross-validation of a new procedure for early screening of smoking cessation medications in humans. ClinPharmacolTher 88:109-114.

Perkins KA, Mercinavage M, Fonte CA, Lerman C (2010b) Varenicline’s effects on acute smoking behavior and reward and their association with subsequent abstinence. Psychopharmacology 210:45-51.

Perkins KA (2012) Subjective reactivity to smoking cues as a predictor of quitting success. Nicotine Tob Res 14:383-387.

Piper ME, Piasecki TM, Federman EB, Bolt DM, Smith SS, Fiore MC, Baker TB.(2004) A multiple motives approach to tobacco dependence: the Wisconsin Inventory of Smoking Dependence Motives (WISDM-68). J Consult Clin Psychol. 72(2):139-54.

Rukstalis M, Jepson C, Strasser A, Lynch KG, Perkins K, Patterson F, Lerman C (2005) Naltrexone reduces the relative reinforcing value of nicotine in a cigarette smoking choice paradigm. Psychopharmacology 180:41-48.

Rustin TA,Tate JC (1993) Measuring the stages of change in cigarette smokers*.* J Subst Abuse Treat 10:209-220.

Sayette MA, Hufford MR (1994) Effects of cue exposure and deprivation on cognitive resources in smokers.J AbnPsychol103:812-818.

Scherma M, Panlilio LV, Fadda P et al. (2008)Inhibition of anandamide hydrolysis by cyclohexylcarbamic acid 3'-carbamoyl-3-yl ester (URB597) reverses abuse-related behavioral and neurochemical effects of nicotine in rats. J PharmacolExpTher 327:482-490.

Sibbald B, Roberts C (1998) Understanding controlled trials: crossover trials. BMJ 316:1719.

Simons JS and Gaher RM.The distress tolerance scale: development and validation of a self-report measure. Motivation and Emotion, 2005: 29(2), 83-102.

Singleton EG, Trotman AJ, Zavahir M, Taylor RC, Heishman SJ (2002) Determination of the reliability and validity of the Marijuana Craving Questionnaire using imagery scripts. ExpClinPsychopharmacol 10:47-53.

Singleton EG, Anderson LM, Heishman SJ (2003) Reliability and validity of the Tobacco Craving Questionnaire and validation of a craving-induction procedure using multiple measures of craving and mood.Addiction 98:1537-1546.

Spek V, Lemmens F, Chatrou M, van Kempen S, Pouwer F, Pop V. (2012) Development of a Smoking Abstinence Self-efficacy Questionnaire. Int J Behav Med. [Epub ahead of print]

Spielberger C. D. Psychological determinants of smoking behavior (1986) In R.D. Tollison (Ed.), Smoking and society: toward a more balanced assessment, Heath, Lexington, MA (1986), pp. 89–134

Taylor RC, Harris NA, Singleton EG, Moolchan ET, Heishman SJ (2000) Tobacco craving: intensity-related effects of imagery scripts in drug abusers. ExpClinPsychopharmacol 8:75-87.

U.S. Department of Health and Human Services (1988) The health consequences of smoking: Nicotine addiction. A report of the Surgeon General. Washington DC: U.S. Government Printing Office.

van den Putte B, Yzer M, Willemsen MC, de Bruijn GJ. (2009)The effects of smoking self-identity and quitting self-identity on attempts to quit smoking. HealthPsychol28(5):535-44.

Waters AJ, Shiffman S, Bradley BP, Mogg K (2003) Attentional shifts to smoking cues in smokers.Addiction 98:1409-1417.

The WHOQOL Group. (1994b). The development of the World Health Organization quality of life assessment instrument (the WHOQOL). In J. Orley and W. Kuyken (Eds) Quality of Life Assessment: International Perspectives. Heidelberg: Springer Verlag.

Woods JR, Williams JG, Tavel M (1989) The two-period crossover design in medical research. Ann Int Med 110:560-566.

**Appendix 1**

**Eligibility Checklist for Study Admission**

Inclusion Criteria

___ 19-65 year old males and females

___ smoking at least 10 cigarettes per day for at least 2 years

___ intend to quit smoking within the next 3 months

___ medically and psychologically healthy (see Appendix 2)

Exclusion Criteria

___ currently attempting to quit smoking

___ treatment for tobacco addiction in the past 3 months

___ use of nicotine replacement products, bupropion, or varenicline in the past 3 months as an aid to quit or reduce smoking

___ use of any oral tobacco product in the past 3 months

___ history of drug or alcohol dependence within last 5 years

___ consumption of more than 15 alcoholic drinks per week on average during the past month

___ use of any illicit drug more than once per week on average during the past month

___ current use of gemfibrozil or other fibrate medication

___ current use of any medication that is contraindicated for gemfibrozil or that would interfere with the protocol in the opinion of MAI/QI. This includes, but is not limited to, anticoagulants, statins, other fibrates, other lipid-lowering agents such as niacin or herbal remedies, and any oral or injected medications for diabetes.

___ any pre-existing gall-bladder disease or operation in the past 12 months

___ any history of or current cardiovascular, liver, hepatic or renal disease

___ diabetes

___ pregnant, nursing, or become pregnant during the study

___ use of psychoactive drugs or medications as revealed by urine toxicology

**Appendix 2**

**Screening Criteria for Study Eligibility**

**General Information**

- males and females, 19-65 years old
- no racial or ethnic exclusions

**Drug History**

- smoking at least 10 cigarettes per day for at least 2 years (self-report and urine NicAlert®)
- intend to quit smoking in the next 3 months
- not currently attempting to quit smoking
- no smoking cessation treatment in past 3 months
- no use of nicotine replacement products, bupropion, or varenicline in past 3 months as an aid to quit or reduce smoking
- no history of drug or alcohol dependence within last 5 years
- no more than 15 alcoholic drinks per week on average during the past month
- no use of any illicit drug more than once per week on average during the past month
- no current use of any medication that would interfere with the protocol in the opinion of MAI/Qualified Investigator

**Psychiatric History**

- no history of or current Axis I psychiatric disorders
- no current antidepressant or antipsychotic medication treatment

**Medical History**

Complete history and physical to rule out:

- current pulmonary disease
- history of or current cardiovascular disease
- history of or current hepatic or renal disease
- history of or current gallbladder disease
- history of or current liver disease
- diabetes

Laboratory Tests

## Negative urine pregnancy test

- UrineNicAlert® reading ≥ 3 (urine cotinine level ≥ 100 ng/ml)
- Serum glucose < 200 mg/dl
- Normal urine screen test
- Serum creatinine< 2 mg/dl
- Creatine kinase within normal limits
- CBC within normal limits
- LFTs within normal limits
- EKG within normal limits, with special focus on cQT intervals
- PT/INR within normal limits
- HbA1c within normal limits

**Appendix 3**

**Overview of Study Design**

| **Study phase** | **Activities** |
| --- | --- |
| Assessment | Screening for eligibility |
| Medication visit 1 | Attend clinic to receive medication. Medication start day will be selected to be at least 7 days before the laboratory test days. |
| 1^st^ week of medication | Smoke normally, take medication (gemfibrozil or placebo). Attend clinic for laboratory tests after taking medication for at least one week. Call in daily. |
| 2^nd^ week of medication | Quit attempt week while taking medication. Attend clinic at the end of the week to assess abstinence. Call in daily. |
| Washout week | Participant will not take medication for at least a week. |
| Medication visit 2 | Attend clinic to receive medication. Medication start day will be selected to be at least 7 days before the laboratory test days. |
| 3^rd^ week of medication | Smoke normally, take medication (gemfibrozil or placebo). Attend clinic for laboratory tests after taking medication for at least one week. Call in daily. |
| 4^th^ week of medication | Quit attempt week while taking medication. Attend clinic at the end of the week to assess abstinence. Call in daily. |

**Appendix 4**

**Medication Instruction Form**

You have been given packages that contain study pills. These packages contain enough pills for half the study. You will get more packages for the second half of the study. You will take two capsules each day. The pills contain either gemfibrozil (Lopid®) or are a placebo. You will not know which pills you are taking each day.

**Take two pills every day, one in the morning and one in the evening**

One pill **as outlined in your medication** package is the morning pill (A.M.) and the other is the evening pill (P.M.)**.**

For example, on Day 1, you will take the first pill from the A.M. package.

On the evening of Day 1, take the first pill from the P.M. package.

**How to take the pills**

You should take the pill 30 minutes before a meal and with a full glass (8 ounces) of water.

**Call the lab**

Each time you take your evening pill, call 416 535-8501 ext 30637 and leave a message telling us the time you took the pill. At the evening call, you will answer a few questions about any smoking. You will earn $2 per day for taking the pills, calling us in the evening, and answering the questions.

**If you forget to take a pill**

If it is more than 4 hours before the next pill, take the pill you forgot. If it is less than 4 hours before the next pill, leave the pill you forgot in the package and take the next one.

**Monitoring side effects**

We will ask about side effects each time you visit the clinic. At the end of the second weeks of medication, we will draw some blood to make sure your blood counts, kidney and liver function have not changed from the beginning of the study

If you experience any bad side effects or changes in your mood or behavior, stop taking the pills. Contact Dr. Bernard Le Foll at 416-535-8501 x 34772 during normal business hours or by calling the study pager at 416-232-5254

If you need to seek medical attention for any side effects or other medical problem, tell your physician about your participation in this study and that you might be taking gemfibrozil.

**Return your capsule packages**

At the end of each week, you must bring the pill packages with you to your next visit. Leave any unused capsules in the package.

**Appendix 5**

**Demographic and Smoking History Form**

1. What is your sex?
   1. Male _____
   2. Female _____
2. How old are you? _____
3. How many years of education have you completed? _____
4. What was your income in the last year
   1. < $10,000 _____
   2. $10,000 - $19,999 _____
   3. $20,000 - $29,999 _____
   4. $30,000 - $49,999 _____
   5. $50,000 - $74,999 _____
   6. >$75,000 _____
5. What is your ethnic background?
   1. Hispanic/Latino _____
   2. Not Hispanic/Latino _____
6. What is your racial background?
   1. American Indian/Alaska Native _____
   2. Asian _____
   3. Native Hawaiian or Other Pacific Islander _____
   4. Black or African American _____
   5. White _____
   6. More than one race _____
7. Do you smoke menthol cigarettes? Yes/No
8. On average, how many cigarettes do you smoke each day? _____
9. How long have you been smoking daily? _____
10. How many cigarettes have you smoked today? _____
11. How long ago did you smoke your last cigarette? _____
12. How many times in your life have you seriously tried to quit smoking? _____
13. How many times in the past year have you seriously tried to quit smoking? _____
14. Do you intend to quit smoking in the next month? Yes/No
15. Do you intend to quit smoking in the next 3 months? Yes/No
16. Do you intend to quit smoking in the next 6 months? Yes/No
17. On average, how many times a day do you experience a craving for cigarettes? _____
18. On average, how long does each cigarette craving last? ______
19. How many people who live with you smoke? ____
20. Do you have a significant other (spouse, boyfriend, girlfriend, partner) who smokes? Yes/No

**Appendix 6**

**Fagerström Test for Nicotine Dependence**

1. How soon after you wake up do you have your first cigarette?

A. Within 5 minutes (3)

B. 6-30 minutes (2)

C. 31-60 minutes (1)

D. After 60 minutes (0)

2. Do you find it difficult to refrain from smoking in places where it is forbidden, e.g., in church, at the library, in a cinema, etc.?

A. Yes (1)

B. No (0)

3. Which cigarette would you hate to give up most?

A. The first one in the morning (1)

B. All others (0)

4. How many cigarettes per day do you smoke?

A. 10 or fewer (0)

B. 11-20 (1)

C. 21-30 (2)

D. 31 or more (3)

5. Do you smoke more frequently during the first hours after waking than the rest of the day?

A. Yes (1)

B. No (0)

6. Do you smoke if you are so ill that you are in bed most of the day?

A. Yes (1)

B. No (0)

**Appendix 7**

**Mood Form**

Please indicate how much you are experiencing each of the following moods right now. Use a number from 0 to 6 to indicate how you are feeling.

0 1 2 3 4 5 6

Not Very Somewhat Moderate Much Very Much Extremely

at all slight

Happy_____

Depressed/Blue_____

Joyful_____

Unhappy_____

Pleased_____

Enjoyment/fun_____

Frustrated_____

Worried/Anxious_____

Angry/Hostile_____

**Appendix 8**

**Tobacco Craving Questionnaire**

Circle the horizontal bar that represents your level of craving right now for each of the items below.

1. I would enjoy a cigarette right now.

STRONGLY DISAGREE ____ : ____ : ____ : ____ : ____ : ____ : ____ STRONGLY AGREE

2. If I smoked right now, I would not be able to stop.

STRONGLY DISAGREE ____ : ____ : ____ : ____ : ____ : ____ : ____ STRONGLY AGREE

3. If I had a lit cigarette in my hand, I probably would smoke it.

STRONGLY DISAGREE ____ : ____ : ____ : ____ : ____ : ____ : ____ STRONGLY AGREE

4. A cigarette would taste good right now.

STRONGLY DISAGREE ____ : ____ : ____ : ____ : ____ : ____ : ____ STRONGLY AGREE

5. I would be less irritable now if I could smoke.

STRONGLY DISAGREE ____ : ____ : ____ : ____ : ____ : ____ : ____ STRONGLY AGREE

6. It would be hard to pass up the chance to smoke.

STRONGLY DISAGREE ____ : ____ : ____ : ____ : ____ : ____ : ____ STRONGLY AGREE

7. I could not stop myself from smoking if I had some cigarettes here.

STRONGLY DISAGREE ____ : ____ : ____ : ____ : ____ : ____ : ____ STRONGLY AGREE

8. Smoking a cigarette would be pleasant.

STRONGLY DISAGREE ____ : ____ : ____ : ____ : ____ : ____ : ____ STRONGLY AGREE

9. If I were smoking now I could think more clearly.

STRONGLY DISAGREE ____ : ____ : ____ : ____ : ____ : ____ : ____ STRONGLY AGREE

10. I would not be able to control how much I smoked if I had some cigarettes here.

STRONGLY DISAGREE ____ : ____ : ____ : ____ : ____ : ____ : ____ STRONGLY AGREE

11. I could not easily limit how much I smoked right now.

STRONGLY DISAGREE ____ : ____ : ____ : ____ : ____ : ____ : ____ STRONGLY AGREE

12. I could control things better right now if I could smoke.

STRONGLY DISAGREE ____ : ____ : ____ : ____ : ____ : ____ : ____ STRONGLYAGREE

**Appendix 9**

**Visual Analog Scales (0-100 mm line)**

Place a tick on the line to represent your answer to each of these questions. Ticks to the left are low in score, whereas ticks to the right are high in score.

1. How positive is your mood now?

Not at all Extremely

1. How much do you crave a cigarette now?

Not at all Extremely

1. How negative is your mood now?

Not at all Extremely

1. How much is your urge for a cigarette now?

Not at all Extremely

**Appendix 10**

**Minnesota Nicotine Withdrawal Scale**

Rate yourself based on how you feel right now.

0 = not at all, 1 = slight, 2 = mild, 3 = moderate, 4 = severe

Depressed mood, sad 0 1 2 3 4

Insomnia, sleep problems 0 1 2 3 4

Angry, irritable, frustrated 0 1 2 3 4

Anxious, nervous 0 1 2 3 4

Difficulty concentrating 0 1 2 3 4

Restless, impatient 0 1 2 3 4

Increased appetite, hungry 0 1 2 3 4

**Appendix 11**

**Smoking Contemplation Ladder**

**Appendix 12**

**Modified Cigarette Evaluation Questionnaire**

For each question, please mark the number that best represents how the cigarette you just smoked made you feel.

1 2 3 4 5 6 7

Not Very A little Moderately A lot Quite a lot Extremely

at all little

1. Was smoking satisfying?
2. Did the cigarette taste good?
3. Did you enjoy the sensations in your throat and chest?
4. Did smoking calm you down?
5. Did smoking make you feel more awake?
6. Did smoking make you feel less irritable?
7. Did smoking help you concentrate?
8. Did smoking reduce your hunger for food?
9. Did smoking make you dizzy?
10. Did smoking make you nauseated?
11. Did smoking immediately relieve your craving for a cigarette?
12. Did you enjoy smoking?

**Appendix 13**

**Side Effects Questionnaire**

Rate these effects since your last visit.

0 = not at all, 1 = mild, 2 = moderate, 3 = severe

Dizziness*

Stomach upset*

Memory lapses

Sleepiness*

Irritability

General weakness*

Numbness in fingers or toes*

Muscle or joint pain*

Change in energy level

Tingling skin sensation*

Coughing*

Change in sex drive*

Difficulty swallowing

Abdominal pain*

Change in appetite

Sadness*

Headache*

Anxiety

Blurred vision*

Muscle spasm*

Any bleeding episode*

Bruises*

Jaundice*

Rash*

Inability to sleep

Muscle weakness or tenderness*

Is there any new medical issue since we last saw you?

*potential side effect of gemfibrozil

**Appendix 14**

**Consent Quiz**

**Instructions: Circle either True or False for each question.**

1. One purpose of the study is to see if gemfibrozil (Lopid®) can help people stop smoking.

## True False

2. I will be told which pills are gemfibrozil and which are placebo.

## True False

3. I need to call once a day to inform that I took my pills that day.

## True False

4. The entire study will have two full-day laboratory sessions .

## True False

5. It’s OK to have a few beers the day before experimental test sessions.

## True False

6. The most common side effects of gemfibrozil are upset stomach and abdominal pain.

## True False

7. During 2 weeks of the study, I will have to try to quit smoking.

## True False

8. I will be paid by a check that will be mailed to me 2 weeks after the study.

## True False

9. If I have a bad effect from the pills, I should call the study doctor.

## True False

10. I may not withdraw from the study after I have signed the consent form.

True False

**Appendix 15: Daily Call Materials**

**Instructions for Daily Calls**

**Each evening when you take your medication please call 416 535-8501 ext 30637 after you take your study medication.** This call should be placed immediately following taking your medication. Listen carefully to the message on the phone and follow the instructions. You will answer the questions below.

You will **read** off **each question letter** on the left and **then answer** with **your response**.

| **#** | **Question** | **Possible Answers** |
| --- | --- | --- |
| **A** | Did you take your medication this morning? | Yes No |
| **B** | Did you take your medication this evening? | Yes No |
| **C** | How many cigarettes have you smoked in the past 24 hours? |  |

**Appendix 16**

**Pharmacy Investigational Product Accountability**

Appendix 17

Time Line Follow Back
